# Substance Use and Suicidality in People at Ultra‐High‐Risk of Psychosis: A Systematic Review

**DOI:** 10.1111/eip.70236

**Published:** 2026-07-28

**Authors:** Alicia Tan Jia Hui, Lee D. Mulligan

**Affiliations:** ^1^ Faculty of Biology, Medicine & Health, School of Health Sciences, Division of Psychology & Mental Health University of Manchester Manchester UK

**Keywords:** psychosis, review, substance use, suicide, ultra‐high‐risk (UHR)

## Abstract

**Aim:**

Substance use and suicidality are prevalent among individuals at ultra‐high‐risk (UHR) of psychosis, yet their relationship remains unclear. This systematic review synthesises evidence on the relationship between substance use and suicidality—comprising suicidal ideation, self‐harm, suicide attempts, and suicide deaths—in the UHR population.

**Methods:**

A pre‐registered systematic review (PROSPERO: CRD42024614852) was conducted following the Preferred Reporting Items for Systematic Reviews and Meta‐Analyses (PRISMA) guidelines. Systematic searches of Embase, Medline, PsycINFO, and Web of Science identified studies examining substance use and suicidality in individuals at UHR. Two reviewers independently extracted data and assessed quality using the Mixed Methods Appraisal Tool. Findings were narratively synthesised.

**Results:**

Twelve studies (13 papers; *n* = 2198) met inclusion criteria. Of 40 analyses, only 13 reported significant associations between substance use and suicidality, most of which were derived from a single study. Tobacco use showed the most consistent preliminary associations with suicidal ideation and self‐harm. Alcohol and cannabis were linked to ideation and self‐harm in some analyses, and cocaine to ideation only, particularly when continuous measures were used. In contrast, composite substance use variables were largely unrelated to suicide outcomes. Methodological quality varied, with major limitations including reliance on cross‐sectional data and the use of dichotomous substance use outcomes.

**Conclusion:**

At present, there is insufficient high‐quality evidence to establish a reliable association between substance use and suicidality in individuals at UHR for psychosis. Rigorous longitudinal studies using validated, substance‐specific measures are needed to better understand these relationships and inform risk assessment and early intervention.

## 1 Introduction

The ultra‐high‐risk (UHR) population—also referred to as having an at‐risk mental state (ARMS) or being at clinical high risk for psychosis (CHR‐P)—are help‐seeking individuals identified as being at elevated risk of developing psychosis (Fusar‐Poli 2017; Yung et al. 2005). This risk is typically defined by the presence of one or more prodromal syndromes: attenuated psychotic symptoms, brief limited intermittent psychotic symptoms, or a combination of genetic risk and functional decline (Nelson et al. 2011). While UHR research has historically focused on the risk of transition to psychosis, recent evidence highlights other significant clinical concerns—most notably, high rates of substance use and suicidality (Ang et al. 2025; Buchy et al. 2015; Carney et al. 2017; Taylor et al. 2015).

Substance use is notably prevalent among individuals at UHR for psychosis. A review by Addington et al. (2014) identified cannabis (33%–54%), alcohol (17%–44%), and tobacco (16%–34%) as the most used substances in this group. Consistent with these estimates, El‐Hage et al. (2023) reported that 42% of individuals in a naturalistic UHR cohort used tobacco and 44% used cannabis, further highlighting the substantial prevalence of these substances in this population. In a North American longitudinal study, Buchy et al. (2015) found significantly higher rates of cannabis (23.4% vs. 9.4%) and tobacco use (23.8% vs. 7.2%) among 735 participants at UHR compared to 278 healthy controls, with these patterns persisting over 1 year. Polysubstance use was also common: among those at UHR using cannabis, 75.6% also reported alcohol use and 55.2% reported tobacco use in the past month. Similar trends were observed in an Australian sample, where Carney et al. (2017) reported tobacco use in 52% of individuals at UHR, compared to 45.4% of help‐seeking controls—both well above the national average of 18.6%. Collectively, these findings indicate a consistent pattern of elevated substance use among those at UHR of psychosis, often exceeding rates observed in help‐seeking peers and the general population.

Concurrently, rates of suicidality among individuals at UHR are markedly elevated. A meta‐analysis by Taylor et al. (2015) reported prevalence rates of 66% for recent suicidal ideation, 49% for lifetime self‐harm, and 18% for lifetime suicide attempts. A more recent meta‐analysis focusing on adolescents and youth at UHR (aged ≤ 25 years) also reported high prevalence rates: 58% for lifetime suicidal ideation, 25% for lifetime suicidal attempts, and 56% for current (2‐week) suicidal ideation (Ang et al. 2025). Moreover, Manges et al. (2025) found that recent (2‐week) suicidal thoughts among people at UHR also exceed those observed in individuals with first‐episode or longstanding psychosis. As Pelizza, Forlani, et al. (2025) note, suicidality in this group remains an under‐recognised public health concern that warrants greater attention to associated risk factors.

Substance use disorder is a well‐established risk factor for suicidality, both in the general population and among individuals with psychiatric conditions. Individuals with substance use disorders are estimated to have a 10‐ to 14‐fold increased risk of suicide compared to the general population (Esang and Ahmed 2018). Among those with co‐occurring psychiatric diagnoses, substance use continues to elevate suicide risk. For example, Østergaard et al. (2017) found that any substance use disorder was associated with at least a threefold increase in suicide deaths. Notably, even subclinical substance use has been linked to increased suicidality in individuals with schizophrenia. A meta‐analysis of 50 studies reported significant associations between alcohol use and suicidal ideation, suicide attempts and suicide deaths (Mulligan et al. 2024), while another found that cannabis use was linked with an increased risk of suicide attempts (Mulligan et al. 2025). Despite this extensive evidence, the relationship between substance use and suicidality remains poorly understood in the UHR population—a group situated between the general population and those with established psychotic disorders.

Although both substance use and suicidality are highly prevalent among individuals at UHR for psychosis, their relationship has not been systematically reviewed. Clarifying this relationship could improve suicide risk assessment, inform early intervention strategies, and support the development of targeted policies and prevention efforts. It could also provide a foundation for future research and clinical initiatives focused on reducing substance use and suicidality in this vulnerable group. To address this gap, the present review aimed to synthesise existing evidence on the association between substance use and suicidality in the UHR population. It addressed two key questions: (1) What is the relationship between substance use and suicidality in individuals at UHR of psychosis? and (2) How are specific substances (e.g., tobacco, alcohol, cannabis) related to different forms of suicidality (e.g., suicidal ideation, self‐harm with suicidal intent, suicide attempts, suicide deaths)?

## 2 Methods

A pre‐registered (PROSPERO: CRD42024614852) systematic review was undertaken following guidelines from the Preferred Reporting Items for Systematic Reviews and Meta‐Analyses PRISMA; Page et al. (2021)—see (Appendix A). Prior to registration, a scoping search was undertaken in November 2024 to assess the existing literature and inform the final search strategy.

### 2.1 Search Strategy

Eligible studies were identified through two methods: (1) a systematic search of four bibliographic databases—MEDLINE (OVID interface, 1946 onwards), PsycINFO (OVID interface, 1806 onwards), Embase (OVID interface, 1974 onwards), and Web of Science (Core Collection)—and (2) forward and backward citation searches of included studies. All searches were conducted up to February 2026.

Search terms included: (‘substance use*’ OR ‘substance abuse*’ OR ‘substance misuse*’ OR ‘substance dependen*’ OR ‘substanc*’ OR ‘alcohol*’ OR ‘alcohol use*’ OR ‘alcohol abuse*’ OR ‘alcohol misuse*’ ‘alcohol dependen*’ OR ‘addict*’ OR ‘drug use*’ OR ‘drug abuse*’ OR ‘drug misuse*’ OR ‘drug dependency’ OR ‘cannabis’ OR ‘marijuana’ OR ‘cocaine’ OR ‘heroin’ OR ‘amphetamine*’ OR ‘methamphetamine*’ OR ‘smoking’ OR ‘tobacco’ OR ‘nicotine’).af AND (suicide OR suicid* OR ‘suicidal behavio*’ OR self*harm*).af AND (‘at risk’ OR CAARMS OR prodromal OR ARMS OR ‘ultra‐high‐risk’ OR ‘UHR’ OR prevention AND psychosis or schizo*).af. Medical Subject Headings (MESH) were incorporated to expand the results of the database search to identify all relevant studies.

### 2.2 Study Selection

Studies were included if they met the following criteria: (1) sampled individuals at UHR for psychosis, defined as the presence of attenuated psychotic symptoms, brief limited intermittent psychotic episodes, or trait vulnerability with a marked decline in functioning and aged between 8 and 40 years; (2) included a subjective or objective measure of any substance use (e.g., self‐, informant‐, or clinician‐reported; dichotomous, composite, or continuous; current or past use); (3) assessed suicidality using self‐, informant‐, or clinician‐reported measures (dichotomous or continuous) of suicidal ideation, self‐harm with suicidal intent, suicide attempts, or suicide deaths; and (4) examined the link between substance use and suicidality. Definitions of UHR status, substance use, and suicidality were consistent with established criteria from prior research (Fusar‐Poli et al. 2012; Taylor et al. 2015; Mulligan et al. 2024). All empirical studies using quantitative or qualitative methods were eligible for inclusion. Non‐English publications and conference abstracts were excluded.

Eligibility was assessed in two stages: title and abstract screening, followed by full‐text review. All screening was conducted using EndNote software. First, A.T. screened all titles and abstracts; uncertain cases were retained for full‐text review. Second, A.T. and L.M. independently reviewed all full‐text papers (3 discrepancies out of 105; 97.1% agreement; Cohen's *κ* = 0.785). Discrepancies were resolved through discussion and consensus was achieved.

### 2.3 Data Extraction

Data on study characteristics, sample details, UHR criteria, substance types and measures, outcome measures, and findings were extracted using a standardised form (Appendix B), developed based on the PRISMA checklist (Page et al. 2021) and piloted on an included study. A.T. and L.M. independently extracted all data, which were cross‐checked for accuracy. Neither were blind to the names of authors, institutions, or journals.

### 2.4 Quality Assessment

Risk of bias was assessed using the Mixed Methods Appraisal Tool (MMAT; Hong et al. 2018), chosen for its suitability across diverse study designs. Consistent with MMAT guidelines, no overall score was assigned. Instead, individual criterion ratings were reported to provide detailed quality information. A.T. and L.M. independently assessed quality (8 discrepancies across 84 ratings; 90.5% agreement; Cohen's *κ* = 0.82) and discrepancies were resolved through discussion.

### 2.5 Data Synthesis

Descriptive statistics and relevant analyses were extracted from included studies. For studies lacking statistical analyses, odds ratios and 95% confidence intervals were calculated from 2 × 2 tables using standard methods for dichotomous data (Fleiss and Berlin 2009), where sufficient data were available. Preliminary scoping suggested low potential for meta‐analysis due to limited studies, variability in substances and measures and heterogeneity in study designs. These factors collectively constrained the feasibility of quantitative synthesis.

Therefore, findings were narratively synthesised by substance types, following Popay et al.'s (2005) framework: (1) developing a theoretical understanding of the relationship between substance use and suicidality; (2) producing a preliminary synthesis of findings; (3) exploring relationships within and between studies, including factors explaining variations in effect direction and size; and (4) assessing the robustness of the synthesis based on evidence quality and quantity. Textual description, thematic grouping, and tabulation were used to systematically summarise and interpret the data. Attention was given to identifying patterns, sources of heterogeneity and gaps in knowledge across substance types.

## 3 Results

### 3.1 Study Selection

**FIGURE 1:**
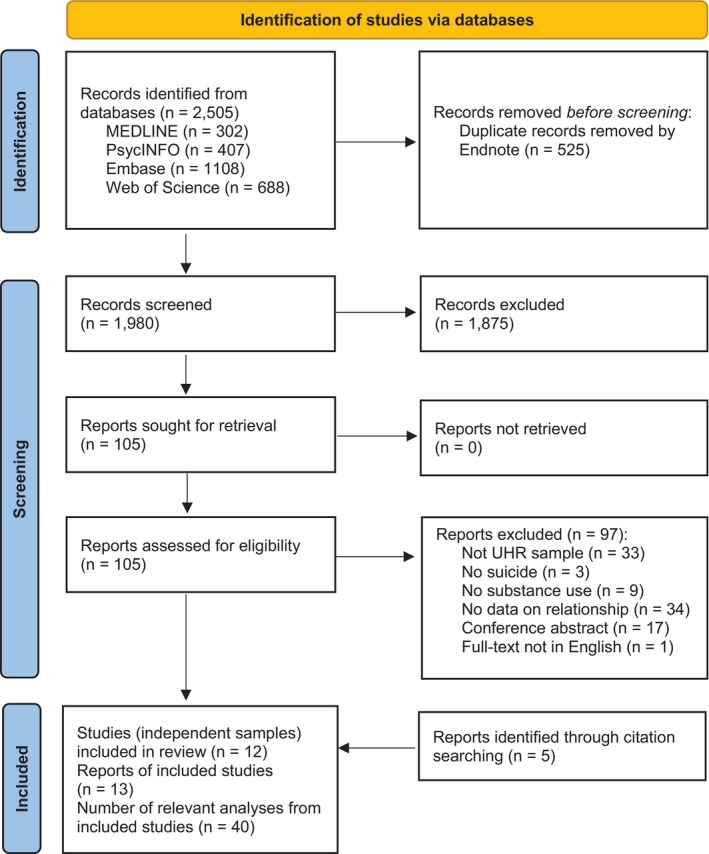
PRISMA flow diagram of study selection.

Figure 1 outlines the study selection process. The systematic search retrieved 2505 records, of which 1980 remained after duplicates were removed. Title and abstract screening excluded 1875 records, leaving 105 for full‐text review. Seven studies (reported in eight papers) met eligibility criteria. An additional five studies were identified through citation tracking, resulting in a total of 12 studies (13 papers) reporting 40 relevant analyses.

### 3.2 Study Characteristics

#### 3.2.1 Sample Source and Size

The 13 papers, published between 2008 and 2025, represented 12 studies from six countries: Greece (Andriopoulos et al. 2011), Ireland (Foley et al. 2008), Italy (Pelizza, Leuci, Quattrone, Azzali, et al. 2024; Pelizza, Leuci, Quattrone, Paulillo, et al. 2024; Pelizza, Di Lisi, et al. 2025; Preti et al. 2009), Tunisia (Fekih‐Romdhane et al. 2023), Turkey (Togay et al. 2015), and the United States (Dobbs et al. 2023; Flanagan and Compton 2012; Pfluger et al. 2025; Trujillo et al. 2025; Zuschlag et al. 2018).

Most studies examined factors associated with suicidality in UHR samples. Seven were cross‐sectional (Andriopoulos et al. 2011; Dobbs et al. 2023; Fekih‐Romdhane et al. 2023; Flanagan and Compton 2012; Foley et al. 2008; Pfluger et al. 2025; Preti et al. 2009), four were longitudinal (Pelizza, Leuci, Quattrone, Azzali, et al. 2024; Pelizza, Leuci, Quattrone, Paulillo, et al. 2024; Pelizza, Di Lisi, et al. 2025; Togay et al. 2015; Trujillo et al. 2025), and one employed a secondary analysis of a pre‐existing database (Zuschlag et al. 2018). Sample sizes ranged from 35 (Fekih‐Romdhane et al. 2023) to 642 (Trujillo et al. 2025) and included a total of 2198 participants across all studies (see Table 1).

**TABLE 1:** Characteristics of included studies.

	Study source (author, year, country)	Study design	Sample source	UHR measure	Substance type and measure (timeframe)	Suicide outcome measure (timeframe)	No. of cases [suicide outcome]	No. of control [no suicide outcome]
1	Andriopoulos et al. (2011), Greece	Cross‐sectional	General University Hospital of Patras, Greece from April 2005 to March 2008	Retrospective interview‐based evaluation for prodromal symptoms	Tobacco use Semi‐structured interview	Suicidal ideation (past month) Semi‐structured interview	27	79
					Alcohol use, abuse or dependence Semi‐structured interview			
						Attempted suicide (past month) Semi‐structured interview	8	98
					Drug use, abuse or dependence Semi‐structured interview			
2	Dobbs et al. (2023) United States	Cross‐sectional	Clinical referrals, self‐referrals, flyers, advertisements through social media and website listings	Structured Interview for Psychosis‐ Risk Syndromes (SIPS)/Scale of Psychosis Risk Syndromes (SOPS)	Substance use disorder Measure not stated	Suicidal ideation (past month) Columbia‐Suicide Severity Rating Scale (C‐SSRS)	10	33
3	Fekih‐Romdhane et al. (2023) Tunisia	Cross‐sectional	Tunisian eaRly Intervention of Psychosis (TRIP) prospective project January 2019 to August 2021	Comprehensive Assessment of At Risk Mental States (CAARMS)	Tobacco use (lifetime) Semi‐structured interview	Attempted suicide (lifetime) Medical records	10	25
					Alcohol use (lifetime) Semi‐structured interview			
					Cannabis use (lifetime) Semi‐structured interview			
4	Flanagan and Compton (2012)	Cross‐sectional	Atlanta Cohort on the Early course of Schizophrenia project from July 2004 to June 2008	Period before first episode psychosis considered as prodromal phase	Alcohol abuse/dependence (lifetime)	Suicidal ideation—in prodromal phase (past 2 weeks) Calgary Depression Scale (CDSS)	24	81
					Cannabis abuse/dependence (lifetime)			
5	Foley et al. (2008)	Cross‐sectional	Dublin East Treatment and Early Care Team (DETECT) from February 2005 to February 2007	Period before first episode psychosis considered as prodromal phase	Alcohol abuse/dependence (lifetime)	Attempted suicide—in prodromal phase Calgary Depression Scale (CDSS)	10	97
					Drug abuse/dependence (lifetime)			
6	Pelizza, Leuci, Quattrone, et al. (2024)^a^ Italy	Longitudinal 2‐year follow‐up period	‘Parma At‐Risk Mental States’ (PARMS) program from January 2016 to December 2020	Comprehensive Assessment of At Risk Mental States (CAARMS)	Substance misuse (current) Study questionnaire	Attempted suicide (lifetime) Study questionnaire	13	138
	Pelizza, Di Lisi, et al. (2025)^a^ Italy					Suicidal ideation (current) Brief Psychiatric Rating Scale (BPRS)	92	88
7	Pelizza, Leuci, Quattrone, et al. (2024) Italy	Longitudinal 1‐year follow‐up period	‘Parma At‐Risk Mental States’ (PARMS) program from January 2013 to December 2017	Comprehensive Assessment of At Risk Mental States (CAARMS)	Substance misuse (current) Study questionnaire	Suicide/self‐harm (past 2 weeks) Health of Nation Outcome Scale (HoNOS)	19	25
8	Pfluger et al. (2025) United States	Cross‐sectional	Center for Early Detection, Assessment, and Response to Risk (CEDAR) from January 2017 and April 2025	Structured Interview for Psychosis‐ Risk Syndromes (SIPS)/Scale of Psychosis Risk Syndromes (SOPS)	Substance use (past 6 months) Alcohol Use Scale (AUS)/Drug Use Scale (DUS)	Suicidal ideation (lifetime) Screening for Risk of Harm (SRH) clinician interview	88	47
						Attempted suicide (lifetime) Screening for Risk of Harm (SRH) clinician interview	30	105
9	Preti et al. (2009) Italy	Cross‐sectional	Programma 2000	Early Recognition Inventory Retrospective Assessment of Symptoms checklist (ERIraos‐CL) and Brief Psychiatric Rating Scale (BPRS)	Substance use Measure not stated	Attempted suicide Assessment interview or clinical records	7	74
					Substance abuse Measure not stated			
10	Togay et al. (2015) Turkey	Longitudinal	First‐episode Schizophrenia Follow‐up Project	Period before first episode psychosis considered as prodromal phase	Alcohol use Measure not stated	Attempted suicide—in prodromal phase Interview with patients and family	29	143
11	Trujillo et al. (2025) United States	Longitudinal	Third sample of the North American Prodrome Longitudinal Study (NAPLS‐3)	Structured Interview for Psychosis‐Risk Syndromes (SIPS)	Tobacco use (lifetime) Structured Clinical Interview for DSM‐IV	Suicidal ideation Calgary Depression Scale (CDSS)	168 (mild) and 42 (mod/high)	474
					Alcohol use and disorder (lifetime) Structured Clinical Interview for DSM‐IV/Alcohol Use Scale (AUS)			
					Marijuana use and disorder (lifetime) Structured Clinical Interview for DSM‐IV/Drug Use Scale (DUS)/Cannabis scale	Self‐harm (lifetime) Structured Assessment of Violence Risk in Youth (SAVRY)	Missing data	Missing data
					Cocaine use (lifetime) Structured Clinical Interview for DSM‐IV/Drug Use Scale (DUS)			
12	Zuschlag et al. (2018) United States	Secondary analysis of a pre‐existing database	Database from Medical University of South Carolina's inpatient and outpatient settings between Jan 2006 and Dec 2010	Diagnostic criteria for attenuated psychosis syndrome (APS) as defined in DSM‐5	Substance abuse Chart review	Attempted suicide (lifetime) Chart review	40	112
					Tobacco use Chart review			

Abbreviations: No., number; UHR, ultra‐high‐risk.

^a^ Pelizza, Leuci, Quattrone, et al. (2024) and Pelizza, Di Lisi, et al. (2025) were treated as a single study, as both drew from the same dataset and data collection period—the Parma At‐Risk Mental States (PARMS) program, conducted from January 2016 to December 2020. Both papers were included in the review as they reported on different suicide‐related outcomes relevant to the review question.

#### 3.2.2 UHR Definition

Participants at UHR for psychosis were identified through three methods: (1) established assessment tools; (2) DSM‐5 criteria; and (3) retrospective evaluation of the prodromal period before schizophrenia onset. Three studies used the Comprehensive Assessment for At‐Risk Mental States (CAARMS; Fekih‐Romdhane et al. 2023; Pelizza, Leuci, Quattrone, Azzali, et al. 2024; Pelizza, Leuci, Quattrone, Paulillo, et al. 2024; Pelizza, Di Lisi, et al. 2025), three used the Structured Interview for Prodromal Syndromes (SIPS; Dobbs et al. 2023; Pfluger et al. 2025; Trujillo et al. 2025), and one combined the Early Recognition Inventory Retrospective Assessment of Symptoms checklist and the Brief Psychiatric Rating Scale (Preti et al. 2009). Zuschlag et al. (2018) adopted the DSM‐5 criteria for attenuated psychosis syndrome. Andriopoulos et al. (2011), Flanagan and Compton (2012), Foley et al. (2008), and Togay et al. (2015) retrospectively assessed suicidality during the prodromal phase in patients with recent‐onset psychosis.

#### 3.2.3 Substance Use

All studies examined substance use; however, there was considerable variation in the substance types, assessment methods and time frames. Three studies examined tobacco use (Andriopoulos et al. 2011; Fekih‐Romdhane et al. 2023; Trujillo et al. 2025) and six assessed alcohol use (Andriopoulos et al. 2011; Fekih‐Romdhane et al. 2023; Flanagan and Compton 2012; Foley et al. 2008; Togay et al. 2015; Trujillo et al. 2025). Two studies examined ‘drug use’ (Andriopoulos et al. 2011; Foley et al. 2008), whereas three focused specifically on cannabis (Fekih‐Romdhane et al. 2023; Flanagan and Compton 2012; Trujillo et al. 2025) and one on cocaine (Trujillo et al. 2025). Six studies included composite measures of ‘substance use’ (Dobbs et al. 2023; Pfluger et al. 2025; Pelizza, Leuci, Quattrone, Azzali, et al. 2024; Pelizza, Leuci, Quattrone, Paulillo, et al. 2024; Pelizza, Di Lisi, et al. 2025; Preti et al. 2009; Zuschlag et al. 2018).

Substance use was often assessed through semi‐structured interviews and scrutiny of medical records (Andriopoulos et al. 2011; Fekih‐Romdhane et al. 2023; Flanagan and Compton 2012; Foley et al. 2008; Pelizza, Leuci, Quattrone, Azzali, et al. 2024; Pelizza, Leuci, Quattrone, Paulillo, et al. 2024; Pelizza, Di Lisi, et al. 2025; Zuschlag et al. 2018). Only two studies used standardised tools—the Alcohol Use Scale (AUS) and Drug Use Scale (DUS) (Pfluger et al. 2025; Trujillo et al. 2025)—with Trujillo et al. (2025) also employing a Cannabis Use Scale. In three studies, the methods used to measure substance use were unclear (Dobbs et al. 2023; Preti et al. 2009; Togay et al. 2015). Most studies reported substance use as a dichotomous variable (present/absent), while Pfluger et al. (2025) and Trujillo et al. (2025) provided mean scores of alcohol and drug use.

Substance use was most assessed over a lifetime time frame (Fekih‐Romdhane et al. 2023; Flanagan and Compton 2012; Foley et al. 2008; Trujillo et al. 2025). Fewer studies assessed current use (Pelizza, Leuci, Quattrone, Azzali, et al. 2024; Pelizza, Leuci, Quattrone, Paulillo, et al. 2024; Pelizza, Di Lisi, et al. 2025) or substance use within the past 6 months (Pfluger et al. 2025). Five studies did not specify the time frame for substance use assessment (Andriopoulos et al. 2011; Dobbs et al. 2023; Preti et al. 2009; Togay et al. 2015; Zuschlag et al. 2018).

#### 3.2.4 Suicidality

Regarding suicidality, six studies examined suicidal ideation (Andriopoulos et al. 2011; Dobbs et al. 2023; Flanagan and Compton 2012; Pelizza, Di Lisi, et al. 2025; Pfluger et al. 2025; Trujillo et al. 2025), two focused on self‐injury or self‐harm with suicidal intent (Pelizza, Leuci, Quattrone, Paulillo, et al. 2024; Trujillo et al. 2025) and eight addressed suicide attempts (Andriopoulos et al. 2011; Fekih‐Romdhane et al. 2023; Foley et al. 2008; Pelizza, Leuci, Quattrone, Azzali, et al. 2024; Pfluger et al. 2025; Preti et al. 2009; Togay et al. 2015; Zuschlag et al. 2018). No studies reported on suicide deaths.

Suicidal ideation and suicide attempts were assessed using semi‐structured interviews and medical records (Andriopoulos et al. 2011; Fekih‐Romdhane et al. 2023; Pelizza, Leuci, Quattrone, Azzali, et al. 2024; Pfluger et al. 2025; Preti et al. 2009; Zuschlag et al. 2018). Only six studies used standardised assessment tools: suicidal ideation was measured using the Brief Psychiatric Rating Scale (BPRS; Pelizza, Di Lisi, et al. 2025), the Calgary Depression Scale for Schizophrenia (CDSS; Flanagan and Compton 2012; Foley et al. 2008; Trujillo et al. 2025), and the Columbia‐Suicide Severity Rating Scale (C‐SSRS; Dobbs et al. 2023); whereas self‐harm was assessed using the Health of the Nation Outcome Scale (HoNOS; Pelizza, Leuci, Quattrone, Paulillo, et al. 2024) and the Structured Assessment of Violence Risk in Youth (SAVRY; Trujillo et al. 2025).

Suicidality was most assessed over a lifetime time frame (Fekih‐Romdhane et al. 2023; Pelizza, Leuci, Quattrone, Azzali, et al. 2024; Pfluger et al. 2025; Trujillo et al. 2025; Zuschlag et al. 2018). Fewer studies examined current suicidality (Pelizza, Di Lisi, et al. 2025), suicidality in the past week (Pelizza, Leuci, Quattrone, Paulillo, et al. 2024), 2 weeks (Flanagan and Compton 2012), or in the past month (Andriopoulos et al. 2011; Dobbs et al. 2023). Three studies did not specify the time frame for suicidality assessment (Foley et al. 2008; Preti et al. 2009; Togay et al. 2015).

### 3.3 Study Quality Assessment

All 12 studies were appraised using the MMAT, with item‐level ratings in Table 2 and details in Appendix C. While all investigated suicidality and associated factors in individuals at UHR, none specifically examined the relationship between substance use and suicidality. Consequently, all studies received a ‘can't tell’ rating for sample representativeness due to insufficient subgroup data on those using substances and non‐users; they reported characteristics only for the overall UHR sample.

**TABLE 2:** Overview of assessment of study methodological quality.

Study (author, year)	Screening questions		Questions specific to quantitative descriptive study design				
	S1. Are there clear research questions?	S2. Do the collected data allow to address the research questions?	4.1. Is the sampling strategy relevant to address the research question?	4.2. Is the sample representative of the target population?	4.3. Are the measurements appropriate?	4.4. Is the risk of nonresponse bias low?	4.5. Is the statistical analysis appropriate to answer the research question?
Andriopoulos et al. (2011)	Yes	Yes	Yes	Can't tell	No	Yes	Can't tell
Dobbs et al. (2023)	Can't tell	Yes	Yes	Can't tell	No	Yes	Can't tell
Fekih‐Romdhane et al. (2023)	Yes	Yes	Yes	Can't tell	No	No	Can't tell
Flanagan and Compton (2012)	Yes	Yes	Yes	Can't tell	No	No	Yes
Foley et al. (2008)	Yes	Yes	Yes	Can't tell	No	Yes	Yes
Pelizza, Leuci, Quattrone, et al. (2024) and Pelizza, Di Lisi, et al. (2025)	Yes	Yes	Yes	Can't tell	No	No	Can't tell
Pelizza, Leuci, Quattrone, et al. (2024)	Yes	Yes	Yes	Can't tell	No	No	Can't tell
Pfluger et al. (2025)	Yes	Yes	Yes	Can't tell	Can't tell	Yes	Can't tell
Preti et al. (2009)	Yes	Yes	Yes	Can't tell	No	Yes	Yes
Togay et al. (2015)	Yes	Yes	Yes	Can't tell	No	Yes	Yes
Trujillo et al. (2025)	Yes	Yes	Yes	Can't tell	Yes	Yes	Yes
Zuschlag et al. (2018)	Yes	Yes	Yes	Can't tell	No	Yes	Yes

Substance use assessment was a major methodological weakness in most studies. Ten studies employed unvalidated or purely diagnostic tools (Andriopoulos et al. 2011; Dobbs et al. 2023; Fekih‐Romdhane et al. 2023; Flanagan and Compton 2012; Foley et al. 2008; Pelizza, Leuci, Quattrone, Azzali, et al. 2024; Pelizza, Leuci, Quattrone, Paulillo, et al. 2024; Pelizza, Di Lisi, et al. 2025; Preti et al. 2009; Togay et al. 2015; Zuschlag et al. 2018), including three studies that did not specify how substance use was assessed (Dobbs et al. 2023; Preti et al. 2009; Togay et al. 2015). Only two studies used standardised tools (Pfluger et al. 2025; Trujillo et al. 2025). However, Pfluger et al. (2025) lacked clarity in reporting, using both AUS and DUS scales but presenting only AUS results. It was unclear whether the reported outcomes for substance use disorders and substance use scale were derived from DUS alone or both AUS and DUS combined, leading to a ‘can't tell’ rating for this item.

Though relevant descriptive statistics were provided, analyses directly addressing the substance use–suicidality relationship were often missing. Six studies received a ‘can't tell’ rating for the appropriateness of their statistical analysis in relation to the review question (Andriopoulos et al. 2011; Fekih‐Romdhane et al. 2023; Dobbs et al. 2023; Pelizza, Leuci, Quattrone, Azzali, et al. 2024; Pelizza, Leuci, Quattrone, Paulillo, et al. 2024; Pelizza, Di Lisi, et al. 2025; Pfluger et al. 2025).

### 3.4 Substance Use and Suicidality

Table 3 summarises 40 analyses from 12 studies on associations between substance use—tobacco, alcohol, drugs, and general substance categories—and suicide‐related outcomes, including suicidal ideation, self‐harm and suicide attempts. Findings were grouped thematically by substance type, with suicide‐related outcomes discussed within each category.

**TABLE 3:** Overview of studies comparing suicidality between substance use and comparison group.

	Study (author, year)	Substance	Suicide outcome	Descriptive statistics [extracted from paper]	Analysis [extracted from paper]	OR (95% CI) [calculated by author]	Conclusion [summarised by author]
1	Andriopoulos et al. (2011)	Tobacco use	Suicidal ideation	1 of 27 who do not smoke had suicidal ideation 26 of 79 who smoked had suicidal ideation	Binomial logistic regression analysis: OR = 14.5 (95% CI: 1.30–166.70) (95% CI: 1.04–4.93) *p* < 0.05		Tobacco use was significantly associated with suicidal ideation after controlling for impairment in concentration, sleep disturbance, lack of appetite and mood swings.
			Suicide attempt	0 of 27 who do not smoke had suicide attempts 8 of 79 who smoked had suicide attempts	Nil	OR = 6.54 (95% CI: 0.36–117.17) *p* = 0.20	Tobacco use was not significantly associated with suicide attempt.
		Alcohol abuse/dependence (exclude occasional alcohol use)	Suicidal ideation	18 of 74 who did not, or occasionally consumed alcohol had suicidal ideation 9 of 32 with alcohol abuse or dependence had suicidal ideation	Nil	OR = 1.22 (95% CI: 0.48–3.10) *p* = 0.68	Alcohol abuse was not significantly associated with suicidal ideation.
			Suicide attempt	4 of 74 who did not, or occasionally consumed alcohol had suicide attempt 4 of 32 with alcohol abuse or dependence had suicide attempt	Nil	OR = 2.5 (95% CI: 0.58–10.70) *p* = 0.22	Alcohol abuse was not significantly associated with suicide attempt.
		Illicit drug abuse and dependence (exclude occasional drug use)	Suicidal ideation	19 of 78 who did not, or occasionally consumed drugs had suicidal ideation 8 of 28 with drug abuse or dependence had suicidal ideation	Nil	OR = 1.24 (95% CI: 0.47–3.27) *p* = 0.66	Drug abuse was not significantly associated with suicidal ideation.
			Suicide attempt	6 of 78 who did not, or occasionally consumed drug had suicide attempt 2 of 28 with drug abuse or dependence had suicide attempt	Nil	OR = 0.92 (95% CI: 0.18–4.86) *p* = 0.92	Drug abuse was not significantly associated with suicide attempt.
2	Dobbs et al. (2023)	Substance use disorder	Suicidal ideation	7 of 34 without substance use disorder had suicide ideation. 3 of 9 with substance use disorder had suicide ideation.	Nil	OR = 1.93 (95% CI: 0.38–9.71) *p* = 0.43	Substance use disorder was not significantly associated with suicidal ideation.
3	Fekih‐Romdhane et al. (2023)	Tobacco use	Suicide attempt	8 of 27 without tobacco consumption had suicide attempt 2 of 8 with tobacco consumption had suicide attempt	*χ* ^*2*^ = 0.07 *p* = 0.80		Tobacco use was not significantly associated with suicide attempt.
		Alcohol use		8 of 29 without alcohol use had suicide attempt 2 of 6 with alcohol use had suicide attempt	*χ* ^*2*^ = 0.08 *p* = 0.78		Alcohol use was not significantly associated with suicide attempt.
		Cannabis use		8 of 31 without cannabis use had suicide attempts 2 of 4 with cannabis use had suicide attempt	*χ* ^*2*^ = 1.02 *p* = 0.56		Cannabis use was not significantly associated with suicide attempt.
4	Flanagan and Compton (2012)	Alcohol abuse/dependence	Suicidal ideation (CDSS)	Data missing from paper	*β* = −1.12 Wald = 2.18 *p* = 0.14		Alcohol abuse/dependence was not significantly associated with suicidal ideation.
		Cannabis abuse/dependence		Data missing from paper	*β* = 0.21 Wald = 0.13 *p* = 0.72		Cannabis abuse/dependence was not significantly associated with suicidal ideation.
5	Foley et al. (2008)	Alcohol abuse/dependence	Suicide attempt (CDSS)	Data missing from paper	*β* = −0.11 Wald = 0.01 *p* = 0.89		Alcohol abuse/dependence was not significantly associated with suicide attempt.
		Drug abuse/dependence		Data missing from paper	*β* = 0.26 Wald = 0.07 *p* = 0.90		Drug abuse/dependence was not significantly associated with suicide attempt.
6	Pelizza, Leuci, Quattrone, et al. (2024)^a^	Current substance misuse (at entry)	Suicide attempt	12 of 123 without substance misuse had suicide attempt 1 of 28 with substance misuse had suicide attempt	HR = 0.374 (95% CI: 0.05–2.88) *p* = 0.35		Current substance misuse was not significantly associated with suicide attempt.
	Pelizza, Di Lisi, et al. (2025)^a^		Suicidal ideation (BPRS)	80 of 149 without substance misuse had suicidal ideation 12 of 31 with substance misuse had suicidal ideation	*Χ* ^2^ = 2.31 *p* = 0.13		Current substance misuse was not significantly associated with suicidal ideation.
7	Pelizza, Leuci, Quattrone, et al. (2024)	Current substance misuse (at entry)	Non‐accidental self‐injury (HoNOS)	14 of 34 without substance misuse had suicide/self‐harm 5 of 10 with substance misuse had suicide/self‐harm	*Χ* ^2^ = 0.25 *p* = NS		Current substance misuse was not significantly associated with self‐injury.
8	Pfluger et al. (2025)	Substance use disorder	Lifetime clinician recorded responses of suicidal ideation	75 of 117 without substance use disorder had suicidal ideation 13 of 18 with substance use disorder had suicidal ideation	*X* ^*2*^ = 0.49 *p* = 0.48		Substance use disorder was not significantly associated with suicidal ideation.
			Lifetime responses of suicide attempt	24 of 118 without substance use disorder had suicide attempt 6 of 17 with substance use disorder had suicide attempt	*X* ^*2*^ = 1.78 *p* = 0.18		Substance use disorder was not significantly associated with suicide attempt.
		Alcohol mean score	Lifetime clinician recorded responses of suicidal ideation	Suicidal ideation absent: *M* = 1.2, SD = 0.5 Suicidal ideation present: *M* = 1.3, SD = 0.55	*T* = 1.03 *p* = 0.31		Alcohol mean score was not significantly associated with suicidal ideation.
			Lifetime responses of suicide attempt	Suicide attempt absent: *M* = 1.24, SD = 5.34 Suicide attempt present: *M* = 1.33, SD = 0.55	*T* = 0.81 *p* = 0.42		Alcohol mean score was not significantly associated with suicide attempt.
		Substance use mean score	Lifetime clinician recorded responses of suicidal ideation	Suicidal ideation absent: *M* = 1.21, SD = 5.5 Suicidal ideation present: *M* = 1.45, SD = 0.92	*T* = 1.91 *p* = 0.06		Substance use mean score was not significantly associated with suicidal ideation.
			Lifetime responses of suicide attempt	Suicide attempt absent: *M* = 1.28, SD = 0.68 Suicide attempt present: *M* = 1.7, SD = 1.15	*T* = 1.9 *p* = 0.65		Substance use mean score was not significantly associated with suicide attempt.
9	Preti et al. (2009)	Substance use	Past attempted suicide	3 of 60 without substance use had past suicide attempt 4 of 21 with substance use had past suicide attempt	Fisher's exact test *p* = 0.07		Substance use was not significantly associated with suicide attempt.
		Substance abuse		3 of 76 without substance abuse had past suicide attempt 4 of 5 with substance abuse had past suicide attempt	Fisher's exact test *p* < 0.01**		Substance abuse was significantly associated with suicide attempt.
10	Togay et al. (2015)	Alcohol use	Suicide attempt before first admission	Data missing from paper	*β* = −3.23 SE = 1.61 *p* < 0.05*		Alcohol use was significantly associated with suicide attempt.
11	Trujillo et al. (2025)	Tobacco mean score	Suicidal ideation (CDSS)	Suicidal ideation absent: *M* = 1.17, SE = 0.02 Suicidal ideation mild: *M* = 1.32, SE = 0.04 Suicide ideation mod/severe: *M* = 1.29, SE = 0.07	*F* = 6.21 *p* = < 0.01** Mild > Absent		Tobacco mean score was significantly associated with suicidal ideation.
			Self‐harm (SAVRY)	Self‐harm none: *M* = 1.18, SE = 0.02 Self‐harm mod: *M* = 1.27, SE = 0.04 Self‐harm high: *M* = 1.33, SE = 0.06	*F* = 4.57 *p* = < 0.02* Group‐level comparison not specified		Tobacco mean score was significantly associated with self‐harm.
		Alcohol mean score	Suicidal ideation (CDSS)	Suicidal ideation absent: *M* = 1.35, SE = 0.03 Suicidal ideation mild: *M* = 1.52, SE = 0.04 Suicide ideation mod/severe: *M* = 1.38, SE = 0.08	*F* = 5.70 *p* = < 0.01** Mild > Absent		Alcohol mean score was significantly associated with suicidal ideation.
			Self‐harm (SAVRY)	Self‐harm none: *M* = 1.33, SE = 0.03 Self‐harm mod: *M* = 1.47, SE = 0.04 Self‐harm high: *M* = 1.57, SE = 0.06	*F* = 8.29 *p* = < 0.01** Mod and high > none		Alcohol mean score was significantly associated with self‐harm.
		Alcohol use disorder	Suicidal ideation (CDSS)	Suicidal ideation absent: *n* = 8 Suicidal ideation mild: *n* = 10 Suicide ideation mod/severe: *n* = 1	*X* ^*2*^ = 8.21 *p* < 0.02*		Alcohol use disorder was significantly associated with suicidal ideation.
			Self‐harm (SAVRY)	Self‐harm none: *n* = 7 Self‐harm mod: *n* = 8 Self‐harm high: *n* = 4	*X* ^*2*^ = 5.51 *p* = NS		Alcohol use disorder was not significantly associated with self‐harm.
	
		Cannabis mean score	Suicidal ideation (CDSS)	Suicidal ideation absent: *M* = 1.29, SE = 0.03 Suicidal ideation mild: *M* = 1.44, SE = 0.05 Suicide ideation mod/severe: *M* = 1.16, SE = 0.09	*F* = 5.35 *p* = < 0.01** Group‐level comparison not specified		Cannabis mean score was significantly associated with suicidal ideation.
			Self‐harm (SAVRY)	Self‐harm none: *M* = 1.28, SE = 0.03 Self‐harm mod: *M* = 1.31, SE = 0.05 Self‐harm high: *M* = 1.51, SE = 0.07	*F* = 4.82 *p* = < 0.01* High > Mod and none		Cannabis mean score was significantly associated with self‐harm.
		Cannabis use disorder	Suicidal Ideation (CDSS)	Suicidal ideation absent: *n* = 33 Suicidal ideation mild: *n* = 15 Suicide ideation mod/severe: *n* = 1	*X* ^*2*^ = 2.64 *p* = NS		Cannabis use disorder was not significantly associated with suicidal ideation.
			Self‐harm (SAVRY)	Self‐harm none: *n* = 30 Self‐harm mod: *n* = 8 Self‐harm high: *n* = 11	*X* ^*2*^ = 6.71 *p* = < 0.04*		Cannabis use disorder was significantly associated with self‐harm.
		Cocaine mean score	Suicidal ideation (CDSS)	Suicidal ideation absent: *M* = 1.00, SE = 0.01 Suicidal ideation mild: *M* = 1.04, SE = 0.01 Suicidal ideation mod/severe: *M* = 1.00, SE = 0.02	*F* = 4.48 *p* = < 0.02* Mild > Absent		Cocaine mean score was significantly associated with suicidal ideation.
Self‐harm (SAVRY)	Self‐harm none: *M* = 1.01, SE = 0.01 Self‐harm mod: *M* = 1.02, SE = 0.01 Self‐harm high: *M* = 1.00, SE = 0.01		*F* = 1.19 *p* = NS		Cocaine mean score was not significantly associated with self‐harm.
12	Zuschlag et al. (2018)	Substance abuse	Lifetime suicide attempts	14 of 61 without substance abuse had lifetime suicide attempts 25 of 91 with substance abuse had lifetime suicide attempts	*X* ^*2*^ = missing data *p* = 0.44	OR = 1.27 (95% CI: 0.60–2.70) *p* = 0.53	Substance abuse was not significantly associated with suicide attempt.
		Tobacco use		16 of 82 without tobacco use disorder had lifetime suicide attempts 24 of 68 with tobacco use disorder had lifetime suicide attempts	*X* ^*2*^ = missing data *p* = 0.03* Tobacco use as risk factor for attempted suicide: *p* = 0.03* Multivariable logistic regression analysis: OR = 2.27 (95% CI: 1.04–4.93) *p* = 0.04*		Tobacco use was significantly associated with suicide attempt after controlling for race, sex and age.

Abbreviations: BPRS, Brief Psychiatric Rating Scale; CDSS, Calgary Depression Scale; CI, confidence interval; *F*, *f*‐statistic; HoNOS, Health of Nation Outcome Scale; HR, hazard ratio; *M*, mean; Mod, moderate; NS, not significant; OR, odds ratio; *p*, probability; SAVRY, Structured Assessment of Violence Risk in Youth; SD, standard deviation; SE, standard error; *T*, *t*‐statistic; *χ* ^*2*^, chi‐square.

^a^ Pelizza, Leuci, Quattrone, et al. (2024) and Pelizza, Di Lisi, et al. (2025) were treated as a single study, as both drew from the same dataset and data collection period—the Parma At‐Risk Mental States (PARMS) program, conducted from January 2016 to December 2020. Both papers were included in the review as they reported on different suicide‐related outcomes relevant to the review question.

*p < 0.05, **p < 0.01.

#### 3.4.1 Tobacco Use

Four studies examined tobacco use and suicide‐related outcomes across six analyses: two on suicidal ideation (Andriopoulos et al. 2011; Trujillo et al. 2025), one on self‐harm (Trujillo et al. 2025) and three on suicide attempts (Andriopoulos et al. 2011; Fekih‐Romdhane et al. 2023; Zuschlag et al. 2018). Four analyses reported significant links (Andriopoulos et al. 2011; Trujillo et al. 2025; Zuschlag et al. 2018).

For suicidal ideation, Trujillo et al. (2025; *n* = 642), found higher tobacco use in groups experiencing mild and high suicidal ideation compared to no ideation (*p* < 0.01; mild > absent). Similarly, Andriopoulos et al. (2011; *n* = 106) reported a significant association between tobacco use and ideation (*p* < 0.05). Trujillo et al. (2025) also observed greater tobacco use in moderate and high self‐harm groups versus no self‐harm (*p* < 0.02), though group‐level comparisons were not specified. For suicide attempts, Zuschlag et al. (2018; *n* = 152) found a significant association between tobacco use and lifetime suicide attempts (*p* = 0.04), while Andriopoulos et al. (2011) and Fekih‐Romdhane et al. (2023; *n* = 35) found no associations.

Overall, findings suggest a potential link between tobacco use and suicidality—particularly suicidal ideation and self‐harm—though these are based on a small number of studies. Results for suicide attempts are mixed, possibly due to sample size and measurement differences.

#### 3.4.2 Alcohol Use

Seven studies assessed alcohol or alcohol use disorders and suicide‐related outcomes across twelve analyses: five on suicidal ideation (Andriopoulos et al. 2011; Flanagan and Compton 2012; Pfluger et al. 2025; Trujillo et al. 2025), two on self‐harm (Trujillo et al. 2025) and five on suicide attempts (Andriopoulos et al. 2011; Fekih‐Romdhane et al. 2023; Foley et al. 2008; Pfluger et al. 2025; Togay et al. 2015). Four analyses reported significant associations (Togay et al. 2015; Trujillo et al. 2025).

Findings for suicidal ideation were mixed. Using continuous and categorical measures, Trujillo et al. (2025) found higher mean alcohol use in groups experiencing mild ideation compared to no ideation (*p* < 0.01; mild > absent), and a significant association between alcohol use disorder and ideation (*p* < 0.02). In contrast, Pfluger et al. (2025; *n* = 135) found no significant differences in mean alcohol use based on clinician‐reported suicidal ideation—possibly due to smaller sample and less structured suicide assessment. Both Andriopoulos et al. (2011) and Flanagan and Compton (2012) also found no significant links between alcohol use disorder, abuse or dependence and suicidal ideation. For self‐harm. Trujillo et al. (2025) reported higher mean alcohol use among participants with moderate and high self‐harm compared to those without (*p* < 0.01; moderate and high > none), but categorical measure of alcohol use disorder showed no significant association. Evidence for suicide attempts was mostly non‐significant. Pfluger et al. (2025) found no mean alcohol use differences based on clinician‐reported suicide attempts. Similarly, categorical analyses found no significant differences in suicide attempts between individuals reporting alcohol use and those reporting no use (Fekih‐Romdhane et al. 2023) or between participants with and without alcohol abuse (Andriopoulos et al. 2011). The exception was Togay et al. (2015); *n* = 172, which found a significant association between alcohol use and suicide attempts (*p* = 0.045).

Overall, evidence linking alcohol use to suicidality in individuals at UHR for psychosis is mixed, with some associations for suicidal ideation and self‐harm but mostly non‐significant results for suicide attempts and categorical data.

#### 3.4.3 Drug Use

Five studies examined drug use—including cannabis and cocaine—and suicide‐related outcomes across 11 analyses: five on suicidal ideation (Andriopoulos et al. 2011; Flanagan and Compton 2012; Trujillo et al. 2025), three on self‐harm (Trujillo et al. 2025) and three on suicide attempts (Andriopoulos et al. 2011; Fekih‐Romdhane et al. 2023; Foley et al. 2008). Only one high‐quality study reported significant links (Trujillo et al. 2025).

For suicidal ideation, Trujillo et al. (2025) found that participants with mild ideation had higher mean cannabis use than those with no or moderate/severe ideation (*p* < 0.01), and higher mean cocaine use than those with no ideation (*p* < 0.01; mild > absent). However, cannabis use disorder was not linked to ideation, nor was cannabis abuse or dependence (Flanagan and Compton 2012). Similarly, Andriopoulos et al. (2011) reported no significant association between drug abuse and ideation. Regarding self‐harm, Trujillo et al. (2025) found higher mean cannabis use in people reporting moderate and high self‐harm versus none (*p* < 0.01; high > moderate and none), with cannabis use disorder significantly associated with self‐harm (*p* < 0.04). Mean cocaine use, however, was not associated with self‐harm. No studies found significant links between drug use and suicide attempts. Both Andriopoulos et al. (2011) and Foley et al. (2008) reported no significant associations between suicide attempts and illicit drug abuse or dependence, and Fekih‐Romdhane et al. (2023) found no significant relationship between cannabis use and suicide attempts.

Overall, evidence linking illicit drug use (particularly cannabis and cocaine) to suicidality in individuals at UHR is mixed. Significant associations were observed in some continuous analyses of suicidal ideation and self‐harm, whereas most categorical and suicide attempt analyses were non‐significant.

#### 3.4.4 Substance Use

Six studies assessed general substance use and suicide‐related outcomes across 11 analyses: four on suicidal ideation (Dobbs et al. 2023; Pelizza, Di Lisi, et al. 2025; Pfluger et al. 2025), one on self‐harm (Pelizza, Leuci, Quattrone, Paulillo, et al. 2024) and six on suicide attempts (Pelizza, Leuci, Quattrone, Azzali, et al. 2024; Pelizza, Di Lisi, et al. 2025; Pfluger et al. 2025; Preti et al. 2009; Zuschlag et al. 2018). Only one analysis reported a significant association (Preti et al. 2009).

Across studies, general substance use was not significantly associated with suicidal ideation or self‐harm. Pelizza, Leuci, Quattrone, et al. (2024); Pelizza, Di Lisi, et al. (2025) found no significant associations between substance misuse at entry and either suicidal ideation or non‐accidental self‐injury. Similarly, Dobbs et al. (2023) and Pfluger et al. (2025) reported no significant relationship between suicidal ideation and substance use disorder or mean substance use. Most findings on suicide attempts were also non‐significant, except Preti et al. (2009), who found a significant association between substance abuse and past suicide attempts (*p* < 0.01), though not with substance use. In contrast, Zuschlag et al. (2018) and Pelizza, Leuci, Quattrone, et al. (2024) found no significant associations between substance abuse or misuse and lifetime suicide attempts. Pfluger et al. (2025) also reported no significant association with suicide attempts across both categorical and continuous measures of substance use.

Overall, the evidence linking general substance use to suicidality is weak, with only one significant finding across 11 analyses. This could reflect the broad and vague nature of ‘substance use’ and variation in measurement across studies.

## 4 Discussion

To our knowledge, this is the first systematic review focused specifically on the relationship between substance use and suicidality in individuals at UHR for psychosis. Only 12 studies (13 papers) met inclusion criteria, and notably, none were explicitly designed to examine this relationship. Instead, substance use was typically included as a secondary variable within broader studies of suicide risk. As such, most eligible studies were characterised by significant methodological limitations. Of 40 analyses, only 13 (from five studies) reported significant associations (Andriopoulos et al. 2011; Preti et al. 2009; Togay et al. 2015; Trujillo et al. 2025; Zuschlag et al. 2018). Notably, nine of these were reported in a single high‐quality cohort (Trujillo et al. 2025), with limited replication across other samples. This is surprising as substance use has been consistently associated with suicide‐related outcomes in the general population (Esang and Ahmed 2018) and in other clinical groups, including schizophrenia (Mulligan et al. 2024, 2025; Østergaard et al. 2017).

Among the substances examined, tobacco use showed the most preliminary evidence of associations with suicidality—particularly suicidal ideation and self‐harm—though links to suicide attempts were inconsistent. Alcohol use showed some associations with ideation and self‐harm, especially in a large‐sample study using continuous measures, but findings were mixed across other studies. Cannabis showed limited evidence of associations with ideation and self‐harm, while cocaine was only associated with ideation in a single study. Composite measures of substance use were largely unrelated to suicide outcomes. Overall, while individual substances—tobacco, alcohol, cannabis, and cocaine—appeared more frequently linked to suicidality than general substance use categories, these findings are preliminary and are largely driven by a single cohort study (Trujillo et al. 2025). Therefore, there is currently insufficient high‐quality evidence to establish reliable associations between substance use and suicidality in individuals at UHR for psychosis.

Several factors have likely contributed to these mixed findings, not least the methodological limitations of eligible studies. First, most studies (10 of 12) reported substance use as a dichotomous variable (i.e., based on presence/absence) and only two studies used validated tools (Pfluger et al. 2025; Trujillo et al. 2025). This represents a significant methodological limitation. Dichotomising substance use fails to capture its severity, frequency, quantity, or pattern, thereby reducing variability, interpretability, and potentially obscuring dose–response relationships with suicidality. Such binary operationalisation may underestimate associations, particularly if suicide‐related outcomes are more strongly linked to heavier, more frequent, or more problematic use rather than mere presence of use, as found in other groups (Na et al. 2021). Furthermore, while half of the studies (6 of 12) explored individual substances (i.e., tobacco, alcohol, cannabis or cocaine), the other half examined composite measures of ‘substance use’ that aggregated multiple substances into a single variable. Given the multifaceted nature of substance use, its nuances are often obscured when studies rely on composite measures. Notably, in this review, studies using composite measures were more likely to report consistently non‐significant findings, which may reflect reduced specificity, whereas those examining individual substances appeared more frequently linked to suicidality, albeit these findings are preliminary and not consistently replicated.

Second, all studies relied on self‐reported substance use via interviews, clinical records, or questionnaires covering various time frames (e.g., lifetime, current, past 2 weeks, 6 months, or unspecified). Such variability in reference periods reduces comparability across studies and may contribute to inconsistent findings, as associations with suicidality may differ depending on whether substance use is recent, ongoing, or historical. While convenient and non‐invasive, self‐report methods also raise concerns about validity and reliability, as respondents may underreport or misrepresent use due to social desirability, poor recall, or limited insight (Bharat et al. 2023; Richter and Johnson 2001). Although Bharat et al.'s (2023) meta‐analysis found generally high agreement between self‐reports and biological measures, sensitivity and specificity varied across studies. Consequently, self‐reported substance use, in the absence of biological verification, may be influenced by reporting biases.

Lastly, most studies (7 of 12) relied on cross‐sectional designs, frequently with small sample sizes, which restrict statistical power and preclude conclusions about temporal directionality. Consequently, most reported associations were tested without longitudinal follow‐up or comprehensive multivariable analyses, limiting the ability to rule out reverse causality or adequately adjust for confounding due to shared underlying vulnerabilities (e.g., depressive symptoms or overall clinical severity). Moreover, no eligible studies reported UHR status stratified by sub‐group (e.g., APS, BLIPS, GRD) precluding examination of potential variability in risk across clinically distinct UHR presentations and possibly contributing to the variability and mixed findings identified across studies.

This is the first systematic review to examine the relationship between substance use and suicidality in individuals at UHR for psychosis. Our review has several strengths, including the application of broad inclusion criteria without restrictions on study design or publication date, comprehensive database and citation searching, and a rigorous assessment of methodological quality, enabling a transparent and critical synthesis of the available evidence. However, despite its rigour, several limitations should be noted. First, grey literature was excluded due to challenges in conducting systematic searches and the lack of peer‐review (Dundar et al. 2024). While this may introduce publication bias, the risk is minimal, as the included studies did not focus primarily on the relationship between substance use and suicidality. Citation searching also helped identify additional studies. Second, only English‐language publications were included; however, this had limited impact as only one paper was excluded for language reasons. Third, the small number of studies (*n* = 12) and considerable heterogeneity between studies precluded meta‐analysis. Nonetheless, this review applied broad inclusion criteria without restrictions on study design or publication date, incorporating all available English‐language evidence to date. The 40 analyses offer preliminary insights, though conclusions remain tentative, with some findings based on single studies or analyses.

Taken together, the limited evidence base found in this review highlights several priorities for future research. To strengthen methodological rigour and improve comparability across studies, future investigations should: (1) report baseline differences between users and non‐users within UHR samples; (2) assess specific substances separately rather than aggregating them into broad categories; (3) use standardised, validated tools such as the Alcohol Use Disorders Identification Test (AUDIT; Babor et al. 2001) or the Alcohol, Smoking and Substance Involvement Screening Test (ASSIST; Humeniuk et al. 2010), which elicit data on quantity, frequency, severity, and patterns of use and not just dichotomous outcomes (i.e., absence or presence); (4) examine differences across UHR sub‐groups (e.g., APS, BLIPS, GRD) by stratifying analyses, to determine whether associations between substance use and suicidality vary within the UHR population; and (5) adopt longitudinal designs with repeated assessments to explore temporal ordering and how substance use trajectories relate to suicide‐related outcomes over time, including suicide death. Where feasible, incorporating biological measures could also improve accuracy, particularly in contexts where self‐report may be unreliable (Bharat et al. 2023).

A notable gap in the current literature is the limited attention to polysubstance use and qualitative perspectives. Despite evidence that polysubstance use is prevalent among UHR individuals (Buchy et al. 2015; El‐Hage et al. 2023), none of the included studies examined how combined substance use patterns relate to suicidality. Future research should address this, as concurrent use of multiple substances may confer distinct and elevated risks not captured by single‐substance analyses (Connor et al. 2014, 2023). Likewise, qualitative research remains underutilised. Although there were no restrictions on study design, all 12 studies were quantitative. Interviews and focus group discussions offer an opportunity to explore whether and how substance use relates to suicide‐related outcomes in individuals at UHR for psychosis from a lived experience perspective. These methods could uncover the role of substances and the processes linking them to suicidality (Guest et al. 2021). Additionally, mixed‐method designs combining qualitative and quantitative approaches are increasingly important in mental health research to build a stronger evidence base (Palinkas 2014).

Understanding the relationship between substance use and suicidality is crucial for clinical services supporting individuals at UHR for psychosis. While the link between substance use and suicidality is well‐documented in the general population and other clinical groups (Esang and Ahmed 2018; Mulligan et al. 2025; Østergaard et al. 2017), research focused specifically on UHR samples is limited. This gap is concerning given the high prevalence of both substance use (Buchy et al. 2015; Carney et al. 2017) and suicidality (Ang et al. 2025; Manges et al. 2025) in this group. Both factors may contribute to disengagement from care and poorer long‐term outcomes. Emerging evidence shows that many individuals at UHR face adverse outcomes beyond transition to psychosis (Fusar‐Poli et al. 2020), underscoring the need to broaden early intervention efforts. Addressing substance use and suicidality within these services may be vital to improving outcomes and quality of life for this vulnerable population.

## 5 Conclusion

In conclusion, current research on the relationship between substance use and suicidality among individuals at UHR for psychosis is limited, with few studies explicitly designed to examine this relationship and most relying on cross‐sectional data. Preliminary findings indicate that tobacco, alcohol, cannabis, and cocaine may be associated with suicidal ideation or self‐harm in some samples, particularly when continuous measures are used. However, these observations primarily come from a single study, with limited replication elsewhere. Therefore, at present, there is insufficient high‐quality evidence to establish a reliable association between substance use and suicidality in individuals at UHR and conclusions regarding risk remain tentative.

Substance use is complex, varying by type, frequency, severity, and pattern, and its relationship with suicidality may differ across substances and outcomes. Given the prevalence of substance use and suicidality among individuals at UHR, future studies employing longitudinal designs, validated assessment tools, and nuanced substance‐specific measures are urgently needed to better understand these relationships. Addressing these gaps is crucial for informing risk assessment, prevention strategies, and early intervention efforts.

## Funding

This work was supported by the NIHR Manchester Biomedical Research Centre (NIHR203308).

## Ethics Statement

Ethical approval was not required for this study, as it is a systematic review of previously published literature.

## Consent

The authors have nothing to report.

## Conflicts of Interest

The authors declare no conflicts of interest.

## Data Availability

The data that support the findings of this study are available in the appendices.

## Appendix A PRISMA Checklist

**Table:**

Section and topic	Item #	Checklist item	Location where item is reported
Title			
Title	1	Identify the report as a systematic review.	Page 1
Abstract			
Abstract	2	See the PRISMA 2020 for Abstracts checklist.	See below
Introduction			
Rationale	3	Describe the rationale for the review in the context of existing knowledge.	Pages 4 and 5
Objectives	4	Provide an explicit statement of the objective(s) or question(s) the review addresses.	Page 5
Methods			
Eligibility criteria	5	Specify the inclusion and exclusion criteria for the review and how studies were grouped for the syntheses.	Pages 6–8
Information sources	6	Specify all databases, registers, websites, organisations, reference lists and other sources searched or consulted to identify studies. Specify the date when each source was last searched or consulted.	Page 6
Search strategy	7	Present the full search strategies for all databases, registers and websites, including any filters and limits used.	Page 6
Selection process	8	Specify the methods used to decide whether a study met the inclusion criteria of the review, including how many reviewers screened each record and each report retrieved, whether they worked independently, and if applicable, details of automation tools used in the process.	Page 6 and 7
Data collection process	9	Specify the methods used to collect data from reports, including how many reviewers collected data from each report, whether they worked independently, any processes for obtaining or confirming data from study investigators, and if applicable, details of automation tools used in the process.	Page 7
Data items	10a	List and define all outcomes for which data were sought. Specify whether all results that were compatible with each outcome domain in each study were sought (e.g., for all measures, time points, analyses), and if not, the methods used to decide which results to collect.	Page 7
	10b	List and define all other variables for which data were sought (e.g., participant and intervention characteristics, funding sources). Describe any assumptions made about any missing or unclear information.	Page 7
Study risk of bias assessment	11	Specify the methods used to assess risk of bias in the included studies, including details of the tool(s) used, how many reviewers assessed each study and whether they worked independently, and if applicable, details of automation tools used in the process.	Page 7
Effect measures	12	Specify for each outcome the effect measure(s) (e.g., risk ratio, mean difference) used in the synthesis or presentation of results.	Table 3
Synthesis methods	13a	Describe the processes used to decide which studies were eligible for each synthesis (e.g., tabulating the study intervention characteristics and comparing against the planned groups for each synthesis (item #5)).	Pages 7 and 8
	13b	Describe any methods required to prepare the data for presentation or synthesis, such as handling of missing summary statistics, or data conversions.	Page 7
	13c	Describe any methods used to tabulate or visually display results of individual studies and syntheses.	Page 8
	13d	Describe any methods used to synthesise results and provide a rationale for the choice(s). If meta‐analysis was performed, describe the model(s), method(s) to identify the presence and extent of statistical heterogeneity, and software package(s) used.	Page 8
	13e	Describe any methods used to explore possible causes of heterogeneity among study results (e.g., subgroup analysis, meta‐regression).	—
	13f	Describe any sensitivity analyses conducted to assess robustness of the synthesised results.	—
Reporting bias assessment	14	Describe any methods used to assess risk of bias due to missing results in a synthesis (arising from reporting biases).	Page 7
Certainty assessment	15	Describe any methods used to assess certainty (or confidence) in the body of evidence for an outcome.	Page 8
Results			
Study selection	16a	Describe the results of the search and selection process, from the number of records identified in the search to the number of studies included in the review, ideally using a flow diagram.	Page 8 and Figure 1
	16b	Cite studies that might appear to meet the inclusion criteria, but which were excluded, and explain why they were excluded.	—
Study characteristics	17	Cite each included study and present its characteristics.	Table 1
Risk of bias in studies	18	Present assessments of risk of bias for each included study.	Page 19 and Table 2
Results of individual studies	19	For all outcomes, present, for each study: (a) summary statistics for each group (where appropriate) and (b) an effect estimate and its precision (e.g., confidence/credible interval), ideally using structured tables or plots.	Table 3
Results of syntheses	20a	For each synthesis, briefly summarise the characteristics and risk of bias among contributing studies.	Pages 10–34
	20b	Present results of all statistical syntheses conducted. If meta‐analysis was done, present for each the summary estimate and its precision (e.g., confidence/credible interval) and measures of statistical heterogeneity. If comparing groups, describe the direction of the effect.	Pages 10–34
	20c	Present results of all investigations of possible causes of heterogeneity among study results.	Pages 10–34
	20d	Present results of all sensitivity analyses conducted to assess the robustness of the synthesised results.	Pages 10–34
Reporting biases	21	Present assessments of risk of bias due to missing results (arising from reporting biases) for each synthesis assessed.	—
Certainty of evidence	22	Present assessments of certainty (or confidence) in the body of evidence for each outcome assessed.	—
Discussion			
Discussion	23a	Provide a general interpretation of the results in the context of other evidence.	Pages 35–39
	23b	Discuss any limitations of the evidence included in the review.	Pages 35–39
	23c	Discuss any limitations of the review processes used.	Page 37
	23d	Discuss implications of the results for practice, policy and future research.	Pages 38 and 39
Other information			
Registration and protocol	24a	Provide registration information for the review, including register name and registration number, or state that the review was not registered.	Pages 3 and 6
	24b	Indicate where the review protocol can be accessed, or state that a protocol was not prepared.	Pages 3 and 6
	24c	Describe and explain any amendments to information provided at registration or in the protocol.	N/A
Support	25	Describe sources of financial or non‐financial support for the review, and the role of the funders or sponsors in the review.	Page 2
Competing interests	26	Declare any competing interests of review authors.	Page 2
Availability of data, code and other materials	27	Report which of the following are publicly available and where they can be found: template data collection forms; data extracted from included studies; data used for all analyses; analytic code; any other materials used in the review.	Appendices B and C

### PRISMA Abstract Checklist

**Table:**

Section and topic	Item #	Checklist item	Reported (yes/no)
Title			
Title	1	Identify the report as a systematic review.	Y
Background			
Objectives	2	Provide an explicit statement of the main objective(s) or question(s) the review addresses.	Y
Methods			
Eligibility criteria	3	Specify the inclusion and exclusion criteria for the review.	N
Information sources	4	Specify the information sources (e.g., databases, registers) used to identify studies and the date when each was last searched.	Y
Risk of bias	5	Specify the methods used to assess risk of bias in the included studies.	Y
Synthesis of results	6	Specify the methods used to present and synthesise results.	Y
Results			
Included studies	7	Give the total number of included studies and participants and summarise relevant characteristics of studies.	Y
Synthesis of results	8	Present results for main outcomes, preferably indicating the number of included studies and participants for each. If meta‐analysis was done, report the summary estimate and confidence/credible interval. If comparing groups, indicate the direction of the effect (i.e., which group is favoured).	Y
Discussion			
Limitations of evidence	9	Provide a brief summary of the limitations of the evidence included in the review (e.g., study risk of bias, inconsistency and imprecision).	Y
Interpretation	10	Provide a general interpretation of the results and important implications.	Y
Other			
Funding	11	Specify the primary source of funding for the review.	Y
Registration	12	Provide the register name and registration number.	Y

## Appendix B Data Extraction Template

**Table:**

Source (author, year, country bibliographic reference)	
Study design	
Sample source (study name, year)	
Inclusion/exclusion criteria	
UHR measure	
UHR sample (number of participants and demographic details)	
Substance use (specify types, measures etc.)	
Outcome measures	
Findings (results applicable to review question, number of participants included in analyses, analyses conducted)	
Quality appraisal (MMAT)	Screening questions (for all types) S1. Are there clear research questions? Rating: Yes/No/Can't tell Comment: S2. Do the collected data allow to address the research questions? Rating: Yes/No/Can't tell Comment: Quantitative descriptive 4.1. Is the sampling strategy relevant to address the research question? Rating: Yes/No/Can't tell Comment: 4.2. Is the sample representative of the target population? Rating: Yes/No/Can't tell Comment: 4.3. Are the measurements appropriate? Rating: Yes/No/Can't tell Comment: 4.4. Is the risk of nonresponse bias low? Rating: Yes/No/Can't tell Comment: 4.5. Is the statistical analysis appropriate to answer the research question? Rating: Yes/No/Can't tell Comment: *Note: Section 4 of the MMAT was selected as all included studies are quantitative descriptive in nature.
Additional notes	

## Appendix C Data Extraction Forms

### Study 1: Andriopoulos et al. (2011)

**Table:**

Source (author, year, country bibliographic reference)	Andriopoulos, I., Ellul, J., Skokou, M., & Beratis, S. (2011). Suicidality in the ‘prodromal’ phase of schizophrenia. *Comprehensive psychiatry*, *52*(5), 479–485. https://doi.org/10.1016/j.comppsych.2010.10.011 Country: Greece
Study design	Cross‐sectional
Sample source (study name, year)	Department of Psychiatry of the Medical School of the General University Hospital of Patras, Greece, from April 2005 through March 2008.
Inclusion/exclusion criteria	Inclusion criteria: − Patients had up to 3 psychotic episodes with an illness duration of 3 years or less, or they had up to 2 relapses with duration of illness of 6 years or less. Exclusion criteria: − Cases with serious medical diseases, neurological disorders, or mental retardation.
UHR measure	Retrospective evaluation for prodromal symptoms and suicidality
UHR sample (number of participants and demographic details)	106 schizophrenia patients admitted to the Department of Psychiatry of the Medical School of the General University Hospital of Patras, Greece − All patients were of Greek descent. − Of the 106 patients admitted in the study, 74 (69.8%) were male. − The mean age of the patients was 29.6 ± 7.5 years, with a range from 18 to 51 years. − The mean duration of psychosis was 2.7 ± 2.2 years. − Eighty‐six of the patients (81.1%) were unmarried; 11 (10.4%), married; 8 (7.5%), divorced; and 1, widowed. − Eighty‐four (79.2%) were living in urban areas; 11 (10.4%), in semiurban areas; and 11 (10.4%), in rural areas. − Nine (8.5%) of the patients had completed university education; 64 (60.4%), high school education level II; 15 (14.2%), high school leveI; and 17 (16%), elementary school. One was uneducated.
Substance use (specify types, measures etc.)	Patients and control subjects were subjected to a semi‐structured interview to determine use of tobacco, alcohol, or illicit substances.
Outcome measures	Patients and control subjects were interviewed for the presence of suicide ideation or suicide attempt retrospectively. Suicide ideation was assessed using the Scale for Suicide Ideation–Worst Point (SSI‐W) − This is exactly the same with the SSI‐C was also used to rate patients' suicide ideation at its worst point during the prodromal phase. − For the assessment of the prodromal stage suicidality, patients were invited to recall the approximate date and circumstances when they experienced the worst suicidal ideation during the prodromal phase. Afterward, they were asked to keep this experience in mind while the interviewer administered the SSI‐W. Suicide attempt was defined as a self‐injurious behaviour according to which the individual reports intent to die. − Suicidality data were classified as follows: (1) no suicide ideation, no attempts; (2) suicide ideation; and (3) suicide attempts. − The data were segregated into 3 periods: (1) the last month before admission (current episode); (2) the past period, from the first psychotic episode until the current evaluation (lifetime after psychosis onset); and (3) the prodromal period.
Findings (results applicable to review question, number of participants included in analyses, analyses conducted)	Smoking was significantly more frequent among the patients with suicide ideation than in those without suicidal ideation (*p* = 0.003). Duration of prodromal phase, alcohol consumption, use of illicit substances, and number of psychotic episodes did not differ significantly between patients with and without suicide ideation. Binomial logistic regression analysis with suicide ideation as dependent variable showed that… and smoking (*χ* ^2^ = 4.6, OR = 14.5, 95% CI: 1.3–166.7) were independent predictors of suicide ideation during the prodromal phase of psychosis after controlling for impairment in concentration, sleep disturbance, lack of appetite and mood swings.
Quality appraisal (MMAT)	Screening questions (for all types) S1. Are there clear research questions? Rating: Yes Comment: The study's aims were clearly articulated, including potential risk factors for suicidal behaviour, which were relevant to the review objective. S2. Do the collected data allow to address the research questions? Rating: Yes Comment: Study included data on substance use and suicidality in prodromal phase. Quantitative descriptive 4.1. Is the sampling strategy relevant to address the research question? Rating: Yes Comment: The study employed non‐probability (convenience) sampling, recruiting 106 schizophrenia patients from a psychiatric department. The sample was appropriate for addressing the review question, as it allowed retrospective examination of suicidal ideation and attempts during the prodromal phase 4.2. Is the sample representative of the target population? Rating: Can't tell Comment: The study provided a clear description of the overall sample of 106 schizophrenia patients; however, details distinguishing substance users and non‐users (controls) were insufficiently described. 4.3. Are the measurements appropriate? Rating: No Comment: Substance use was assessed without the use of validated measurement tools. 4.4. Is the risk of nonresponse bias low? Rating: Yes Comment: Data was provided for all 106 participants. 4.5. Is the statistical analysis appropriate to answer the research question? Rating: Can't tell Comment: The study's research questions only partially aligned with the focus of this review, and statistical analyses were not reported for all substance types.
Additional notes	Nil

### Study 2: Dobbs et al. (2023)

**Table:**

Source (author, year, country bibliographic reference)	Dobbs, M. F., McGowan, A., Selloni, A., Bilgrami, Z., Sarac, C., Cotter, M., Herrera, S. N., Cecchi, G. A., Goodman, M., Corcoran, C. M., & Srivastava, A. (2023). Linguistic correlates of suicidal ideation in youth at clinical high‐risk for psychosis. *Schizophrenia research*, *259*, 20–27. https://doi.org/10.1016/j.schres.2023.03.014 Country: United States
Study design	Cross‐sectional
Sample source (study name, year)	Participants in this study were ascertained from a variety of sources, including clinical referrals, self‐referrals, flyers, advertisements through social media and website listings.
Inclusion/exclusion criteria	Inclusion criteria: − Fluency in English − Help‐seeking − Met criteria for a psychosis‐risk syndrome, using the Structured Interview for Psychosis‐Risk Syndromes/Scale of Psychosis‐Risk Symptoms (SIPS/SOPS). Exclusion criteria: − History of threshold psychosis (determined by the Presence of Psychosis criteria on the SIPS/SOPS) − Any major neurological or medical disorder − IQ < 70 − Current risk for suicidal or violent behaviour
UHR measure	Structured Interview for Psychosis‐Risk Syndromes/Scale of Psychosis‐Risk Symptoms (SIPS/SOPS)
UHR sample (number of participants and demographic details)	43 CHR participants − 10 endorsed suicidal ideation in the past month (CHR + rSI), whereas 33 did not (CHR − rSI). − Preponderance of females in the CHR + rSI group (60% vs. 40%) − The CHR + rSI and CHR − rSI groups were similar in race, ethnicity, age, comorbid diagnoses (depression, anxiety, substance use disorder), positive and negative symptoms and global functioning. − Use of antipsychotics was equivalent (approximately 20%) but there was a greater, albeit non‐significant, use of antidepressants among CHR + rSI individuals.
Substance use (specify types, measures etc.)	Not specified
Outcome measures	The Columbia‐Suicide Severity Rating Scale (C‐SSRS) is a comprehensive assessment that characterises recent and lifetime suicidal ideation, including level (e.g., wish to be dead, with or without intent and/or plan), nature (of intent and plan, if present), and intensity (frequency, duration, controllability, potential deterrents, reasons), as well as the characterisation of suicidal behaviours and attempts. The scale is designed to assess suicidal ideation and behaviour in the past‐month (recent) and over the entire life course (lifetime). The C‐SSRS is included in the PhenX Toolkit for classification of suicidal ideation in adolescents and adults; to date, it has been used in > 400 studies (PubMed). Each case was reviewed in a consensus meeting with an expert in the assessment of suicidal ideation (co‐author MG). The CHR group was stratified in respect to those who endorsed suicidal ideation within the past month (CHR + rSI) versus those who did not (CHR − rSI). CHR individuals with and without suicidal ideation were compared across demographics, symptoms and medications.
Findings (results applicable to review question, number of participants included in analyses, analyses conducted)	While raw data were presented in tables, the study did not provide accompanying analysis or interpretive summary.
Quality appraisal (MMAT)	Screening questions (for all types) S1. Are there clear research questions? Rating: Can't tell Comment: The study's aims were clearly articulated, which was to use natural language processing (NLP) to identify linguistic correlates of recent suicidal ideation in CHR individuals, which could potentially be relevant to the review objective if use of substances was mentioned. S2. Do the collected data allow to address the research questions? Rating: Yes Comment: Study included data on substance use disorder and suicidality. Quantitative descriptive 4.1. Is the sampling strategy relevant to address the research question? Rating: Yes Comment: The study employed non‐probability (convenience) sampling, recruiting 43 CHR participants from a variety of sources, including clinical referrals, self‐referrals, flyers, advertisements through social media and website listings. 4.2. Is the sample representative of the target population? Rating: Can't tell Comment: The study provided a clear description of the overall sample of 43 CHR participants; however, details distinguishing substance users and non‐users (controls) were insufficiently described. 4.3. Are the measurements appropriate? Rating: No Comment: Substance use was assessed without the use of validated measurement tools. Additionally, no details provided on how substance use was assessed. 4.4. Is the risk of nonresponse bias low? Rating: Yes Comment: Data was provided for all 43 CHR participants. 4.5. Is the statistical analysis appropriate to answer the research question? Rating: Can't tell Comment: The study's research question was not the focus of this review, and statistical analyses were not reported for substance use disorder.
Additional notes	Nil

### Study 3: Fekih‐Romdhane et al. (2023)

**Table:**

Source (author, year, country bibliographic reference)	Fekih‐Romdhane, F., Abassi, B., Ghrissi, F., Loch, A. A., Cherif, W., Damak, R., Ellini, S., Hallit, S., & Cheour, M. (2023). Suicide risk among individuals at Ultra‐High Risk (UHR) of psychosis in a developing North African country: A 12‐month naturalistic prospective cohort study from the TRIP project. *Psychiatry research*, *327*, 115 409. https://doi.org/10.1016/j.psychres.2023.115409 Country: Tunisia
Study design	Longitudinal
Sample source (study name, year)	Tunisian eaRly Intervention of Psychosis (TRIP) prospective project at the Tunisian Center of Early Intervention in Psychosis (TCEIP) located at the Razi Psychiatric Hospital, Manouba, Tunisia, from January 2019 to August 2021.
Inclusion/exclusion criteria	Inclusion criteria: − Aged 16–35 years were antipsychotics‐naïve and had no previous diagnosed psychotic disorders as defined according to the Diagnostic and Statistical Manual of Mental Disorders, fifth edition (DSM‐5) American Psychiatric Association and Association, 2013 met the CAARMS‐defined UHR/FEP criteria. Exclusion criteria: − Reported having any serious medical condition (including neurological illness and head injury) had an intellectual disability according to the DSM‐5 criteria.
UHR measure	Comprehensive Assessment of At Risk Mental States (CAARMS)
UHR sample (number of participants and demographic details)	35 UHR participants − 15–35 years of age (mean = 22.8; S.D. = 4.0) − 45.7% male; − 54.3% were self‐referred to the service − 40% indicated that the main reason for help‐seeking was decreased academic/work performance.
Substance use (specify types, measures etc.)	No specific detail besides the line stating that ‘clinical evaluation involved assessments regarding sociodemographic and clinical information, including …substance use’.
Outcome measures	The CAARMS item 7.3 ‘Suicidality/Self Harm’ is a 7‐point component that assesses suicidal ideation and self‐injurious behaviour. A cutoff score of ≥ 2 refers to the presence of at least occasional, passive suicidal or self‐harm ideation, with no active suicidal plan or behaviour (Yung et al. 2005). Lifetime and follow‐up suicide attempts correspond to any self‐inflicted, potentially injurious behaviour with the intent to die, and without a fatal outcome. The number of suicide attempts at baseline, 6 and 12‐months was obtained from information documented in their clinical records.
Findings (results applicable to review question, number of participants included in analyses, analyses conducted)	While raw data were presented in tables, the study did not provide accompanying analysis or interpretive summary.
Quality appraisal (MMAT)	Screening questions (for all types) S1. Are there clear research questions? Rating: Yes Comment: The study's aims were clearly articulated, including potential risk factors related to lifetime suicide attempts (cross‐sectionally) and suicidal ideation (longitudinally) within the UHR group, which were relevant to the review objective. S2. Do the collected data allow to address the research questions? Comment: Yes Comment: Study included data on substance use and suicidality in the UHR group. Quantitative descriptive 4.1. Is the sampling strategy relevant to address the research question? Rating: Yes Comment: The study employed non‐probability (convenience) sampling of help‐seekers at the Tunisian Center of Early Intervention in Psychosis (TCEIP). 4.2. Is the sample representative of the target population? Rating: Can't tell Comment: The study provided a clear description of the overall sample of 35 UHR participants; however, details distinguishing substance users and non‐users (controls) were insufficiently described. 4.3. Are the measurements appropriate? Rating: No Comment: Substance use was assessed without the use of validated measurement tools. Additionally, no details provided on how substance use was assessed. 4.4. Is the risk of nonresponse bias low? Rating: No Comment: Although reasons for excluding 39 of the 74 eligible CHR participants were reported (e.g., missing data, loss to follow‐up, transition to FEP), the study did not provide demographic information or conduct analyses to assess differences between included and excluded participants. 4.5. Is the statistical analysis appropriate to answer the research question? Rating: Can't tell Comment: The study's research questions only partially aligned with the focus of this review, and statistical analyses were not reported for substance types.
Additional notes	Nil

### Study 4: Flanagan and Compton (2012)

**Table:**

Source (author, year, country bibliographic reference)	Flanagan, P., & Compton, M. T. (2012). A comparison of correlates of suicidal ideation prior to initial hospitalisation for first‐episode psychosis with prior research on correlates of suicide attempts prior to initial treatment seeking. *Early intervention in psychiatry*, *6*(2), 138–144. https://doi.org/10.1111/j.1751‐7893.2011.00320.x Country: United States
Study design	Cross‐sectional
Sample source (study name, year)	Atlanta Cohort on the Early course of Schizophrenia project from July 2004 to June 2008
Inclusion/exclusion criteria	Inclusion criteria: − Aged 18–40 years − Current inpatient with FEP Exclusion criteria: − No diagnosis of primary psychotic disorder − Known or suspected diagnosis of mental retardation or Mini Mental State Examination score of < 24 − Having > 3 months of prior antipsychotic treatment − Having been hospitalised for psychosis > 3 months prior to index admission
UHR measure	Note by author: the period before first episode psychosis considered as prodromal phase
UHR sample (number of participants and demographic details)	109 patients with FEP − 76.1% male − 89.9% African American − 91.7% single/never married − Age at hospitalisation (mean = 23.1, SD = 4.7)
Substance use (specify types, measures etc.)	Diagnoses of substance use disorders were made using the Structured Clinical Interview for DSM‐IV Axis I Disorders (SCID), based on all available information gathered through a semi‐structured interview, review of the patient's hospital chart, discussions with treating clinicians and collateral information from family members when available. Alcohol abuse/dependence and cannabis abuse/dependence variables extracted.
Outcome measures	Suicidal ideation was rated with item #8 of the CDSS, which queries about suicidal ideation in the past 2 weeks using the following probe questions: ‘Have you felt that life wasn't worth living? Did you ever feel like ending it all? What did you think you might do? Did you actually try?’ For this analysis, the sample was dichotomized as having a score of 0 (77.1%) versus having suicidal ideation, indicated as a score of 1–3 (22.9%).
Findings (results applicable to review question, number of participants included in analyses, analyses conducted)	Neither alcohol abuse/dependence (*β* = −1.12, *p* = 0.14) nor cannabis abuse/dependence (*β* = 0.21 *p* = 0.72) significantly predicted suicidal ideation in logistic regression analyses with ideation as the outcome variable. Number included in logistic regression (*n* = 24).
Quality appraisal (MMAT)	Screening questions (for all types) S1. Are there clear research questions? Rating: Yes Comment: The study's aims were clearly articulated, to replicate Foley et al. (2008) on predictors of suicidality in prodromal period but with a focus on suicidal ideation. S2. Do the collected data allow to address the research questions? Comment: Yes Comment: Study included data on substance use and suicidality in the UHR group. Quantitative descriptive 4.1. Is the sampling strategy relevant to address the research question? Rating: Yes Comment: The study employed non‐probability (convenience) sampling and recruited participants from inpatient psychiatric units. 4.2. Is the sample representative of the target population? Rating: Can't tell Comment: The study provided a description of the overall sample; however, details distinguishing substance users and non‐users (controls) were insufficiently described. 4.3. Are the measurements appropriate? Rating: No Comment: Substance use was assessed without the use of validated measurement tools. 4.4. Is the risk of nonresponse bias low? Rating: No Comment: Although reasons for excluding 83 of the 192 eligible participants were reported (e.g., missing data, declined participation, discharged from hospital), the study did not provide demographic information or conduct analyses to assess differences between included and excluded participants. 4.5. Is the statistical analysis appropriate to answer the research question? Rating: Yes Comment: The logistic regression analyses calculating the relationship between substance use and suicidal ideation appropriately answers the research question re. correlates of suicidality in the UHR group.
Additional notes	Nil

### Study 5: Foley et al. (2008)

**Table:**

Source (author, year, country bibliographic reference)	Foley, S., Jackson, D., McWilliams, S., Renwick, L., Sutton, M., Turner, N., … & O'Callaghan, E. (2008). Suicidality prior to presentation in first‐episode psychosis. *Early intervention in psychiatry*, *2*(4), 242–246. https://doi.org/10.1111/j.1751‐7893.2008.00084.x Country: Ireland
Study design	Cross‐sectional
Sample source (study name, year)	Dublin East Treatment and Early Care Team (DETECT) from February 2005 to February 2007
Inclusion/exclusion criteria	Inclusion criteria: − Aged 18–40 years − Current diagnosis of FEP − Inpatient or outpatient Exclusion criteria: − Non described
UHR measure	Note by author: the period before first episode psychosis considered as prodromal phase
UHR sample (number of participants and demographic details)	107 patients with FEP − 63% male − 67% inpatient − 87% single/never married
Substance use (specify types, measures etc.)	Information on alcohol and substance abuse was gathered from the SCID‐I interview. Alcohol abuse/dependence and drug abuse/dependence variables extracted.
Outcome measures	Information on past suicide attempts was gathered from the SCID‐I module A section for current (past month) and/or past (ever) depression, and question 8 of the Calgary Depression Scale, where an attempt in the past 2 weeks scores 3. Dates of suicide attempts were clarified from the SCID‐1 interview and any collateral information available.
Findings (results applicable to review question, number of participants included in analyses, analyses conducted)	Neither alcohol abuse/dependence (*β* = −0.11, *p* = 0.89) nor drug abuse/dependence (*β* = 0.26 *p* = 0.90) significantly predicted suicide attempts in logistic regression analyses with suicide attempts as the outcome variable. Number included in logistic regression (*n* = 10).
Quality appraisal (MMAT)	Screening questions (for all types) S1. Are there clear research questions? Rating: Yes Comment: The study's aims were clearly articulated, to assess predictors of suicidality in prodromal period but with a focus on attempted suicide. S2. Do the collected data allow to address the research questions? Comment: Yes Comment: Study included data on substance use and suicidality in the UHR group. Quantitative descriptive 4.1. Is the sampling strategy relevant to address the research question? Rating: Yes Comment: The study employed non‐probability (convenience) sampling and recruited participants seeking help from DETECT (including outpatient and inpatient psychiatric units). 4.2. Is the sample representative of the target population? Rating: Can't tell Comment: The study provided a description of the overall sample; however, details distinguishing substance users and non‐users (controls) were insufficiently described. 4.3. Are the measurements appropriate? Rating: No Comment: Substance use was assessed without the use of validated measurement tools. 4.4. Is the risk of nonresponse bias low? Rating: Yes Comment: Only 16 participants out of 123 had missing data and the reasons are adequately described. 4.5. Is the statistical analysis appropriate to answer the research question? Rating: Yes Comment: The logistic regression analyses calculating the relationship between substance use and suicide attempt appropriately answers the research question re. correlates of suicidality in the UHR group.
Additional notes	Nil

### Study 6a: Pelizza, Leuci, Quattrone, et al. (2024)

**Table:**

Source (author, year, country bibliographic reference)	Pelizza, L., Leuci, E., Quattrone, E., Azzali, S., Pupo, S., Paulillo, G., Menchetti, M., & Pellegrini, P. (2024). Adverse outcome analysis in people at clinical high risk for psychosis: results from a 2‐year Italian follow‐up study. *Social psychiatry and psychiatric epidemiology*, *59*(7), 1177–1191. https://doi.org/10.1007/s00127‐023‐02597‐8 Country: Italy
Study design	Longitudinal across 2‐year follow‐up period
Sample source (study name, year)	‘Parma At‐Risk Mental States’ (PARMS) program between 1st January 2016 and 31st December 2020
Inclusion/exclusion criteria	Inclusion criteria: − Age 12–25 years; − Specialist help‐seeking request; − CHR‐P status, as defined by the ‘Comprehensive Assessment of At‐Risk Mental States’ (CAARMS) criteria: that is, ‘Brief Limited Intermittent Psychotic Symptoms’ (BLIPS), ‘Attenuated Psychotic Symptoms’ (APS) and ‘Genetic Vulnerability’ Exclusion criteria: − History of DSM‐IV‐TR affective or non‐affective psychotic episode; − Past exposure to antipsychotic medication; − Current DSM‐IV‐TR substance dependence; − Known severe/moderate intellectual disability (i.e., IQ < 50); and − Neurological disorder or any other medical condition presenting with psychiatric symptoms.
UHR measure	Comprehensive Assessment of At Risk Mental States (CAARMS)
UHR sample (number of participants and demographic details)	164 UHR participants − 128 (78%) CHR‐P individuals met APS criteria and 30 (18.3%) met BLIPS criteria. − Depressive disorders were the most common DSM‐IV‐TR diagnosis (*n* = 50; 30.5%), followed by schizotypal personality disorder (*n* = 41; 25%). − General practitioners (*n* = 74; 45.1%) were the most frequent source of referral, followed by school/social services (*n* = 31; 18.9%). At baseline, 85 (51.8%) CHR‐P subjects were taking antipsychotics and only 35 (21.3%) were taking antidepressants.
Substance use (specify types, measures etc.)	Sociodemographic/clinical schedule was completed at baseline which collected information on current substance misuse.
Outcome measures	Derived from direct information reported by the subject (or by a relative well informed about the facts) both at baseline and across the 2‐year follow‐up assessment, or documented in the clinical notes. Suicide attempt defined as a potentially injurious, self‐inflicted behaviour without a fatal outcome for which there was (explicit or implicit) evidence of intent to die. Acts of deliberate self‐harm or intoxication with alcohol or drugs, but where there was no clear intention to die, were not considered as suicide attempts.
Findings (results applicable to review question, number of participants included in analyses, analyses conducted)	While raw data were presented in tables, the study did not provide accompanying analysis or interpretive summary.
Quality appraisal (MMAT)	Screening questions (for all types) S1. Are there clear research questions? Rating: Yes Comment: The study's aims were clearly articulated, including specific adverse outcome indicators and association with sociodemographic and clinical features of the CHR‐P sample, which were relevant to the review objective. S2. Do the collected data allow to address the research questions? Comment: Yes Comment: Study included data on substance use and suicidality in the UHR group. Quantitative descriptive 4.1. Is the sampling strategy relevant to address the research question? Rating: Yes Comment: The study employed non‐probability (convenience) sampling of CHR‐P adolescents and young adults in the ‘Parma At‐Risk Mental States’ (PARMS) program between 1st January 2016 and 31st December 2020. 4.2. Is the sample representative of the target population? Rating: Can't tell Comment: The study provided a clear description of the overall sample of 164 CHR‐P participants; however, details distinguishing substance users and non‐users (controls) were insufficiently described. 4.3. Are the measurements appropriate? Rating: No Comment: Substance use was assessed without the use of validated measurement tools. Additionally, no details provided on how substance use was assessed. 4.4. Is the risk of nonresponse bias low? Rating: No Comment: During the follow‐up, 30 (18.3%) CHR‐P individuals dropped out. Kaplan–Meier analysis results confirmed a 2‐year estimated cumulative drop‐out risk of 0.183. Service disengagement was significantly predicted by older age at entry, non‐Caucasian ethnic group, previous specialist contact, baseline presence of BLIPS and brief psychotic disorder, school/social services as source of referral and low acceptance of psychosocial interventions proposed at baseline within the PARMS program. 4.5. Is the statistical analysis appropriate to answer the research question? Rating: Can't tell Comment: The study's research questions only partially aligned with the focus of this review, and statistical analyses were not reported for substance types.
Additional notes	Nil

### Study 6b: Pelizza, Di Lisi, et al. (2025)

**Table:**

Source (author, year, country bibliographic reference)	Pelizza, L., Di Lisi, A., Leuci, E., Quattrone, E., Palmisano, D., Pellegrini, C., Pellegrini, P., Paulillo, G., Pupo, S., & Menchetti, M. (2025). Suicidal thinking and behaviour in young people at Clinical High Risk for Psychosis: Psychopathological considerations and treatment response across a 2‐year follow‐up study. *Suicide & life‐threatening behaviour*, *55*(1), e13136. https://doi.org/10.1111/sltb.13136
Study design	Longitudinal across 2‐year follow‐up period
Sample source (study name, year)	‘Parma At‐Risk Mental States’ (PARMS) program between 1st January 2016 and 31st December 2020
Inclusion/exclusion criteria	Inclusion criteria: − Age 12–25 years; − Specialist help‐seeking request; − CHR‐P status, as defined by the ‘Comprehensive Assessment of At‐Risk Mental States’ (CAARMS) criteria: that is, ‘Brief Limited Intermittent Psychotic Symptoms’ (BLIPS), ‘Attenuated Psychotic Symptoms’ (APS) and ‘Genetic Vulnerability’ Exclusion criteria: − Past psychotic episode; − Previous exposure to AP drug or current AP intake for a duration exceeding 4 weeks in the present illness episode, known intellectual disability (IQ < 70); − Neurological or other medical disorder with psychiatric manifestations
UHR measure	Comprehensive Assessment of At Risk Mental States (CAARMS)
UHR sample (number of participants and demographic details)	180 CHR‐P participants
Substance use (specify types, measures etc.)	Sociodemographic/clinical schedule was completed at baseline which collected information on current substance misuse.
Outcome measures	The presence of current suicidal ideation was detected using an item 4 cut‐ off score of ≥ 3 in the Brief Psychiatric Rating Scale (BPRS) (e.g., ‘Have you felt that life wasn't worth living?’, ‘Have you thought about harming or killing yourself?’, ‘Have you felt tired of living or as though you would be better off dead?’). This cut‐ off score indicated cur‐ rent suicidal ideation. Sociodemographic/clinical chart collecting information on new suicide attempt and self‐ harm.
Findings (results applicable to review question, number of participants included in analyses, analyses conducted)	While raw data were presented in tables, the study did not provide accompanying analysis or interpretive summary.
Quality appraisal	Reder to quality appraisal for 3a.
Additional notes	Nil

### Study 7: Pelizza, Leuci, Quattrone, et al. (2024)

**Table:**

Source (author, year, country bibliographic reference)	Pelizza, L., Leuci, E., Quattrone, E., Paulillo, G., Pupo, S., Pellegrini, P., & Menchetti, M. (2024). Unfavourable short‐term outcome indicators in young people at clinical high risk for psychosis: Preliminary results from the ‘Parma At‐Risk Mental States’ (PARMS) program. *Journal of Psychopathology, 30*(2), 116–130. https://www.jpsychopathol.it/article/view/187 Country: Italy
Study design	Longitudinal across 1‐year follow‐up period
Sample source (study name, year)	‘Parma At‐Risk Mental States’ (PARMS) program between January 2013 and December 2017.
Inclusion/exclusion criteria	Inclusion criteria: − Age 12–25 years; − Specialist help‐seeking request; − CHR‐P status, as defined by the ‘Comprehensive Assessment of At‐Risk Mental States’ (CAARMS) criteria: that is, ‘Brief Limited Intermittent Psychotic Symptoms’ (BLIPS), ‘Attenuated Psychotic Symptoms’ (APS) and ‘Genetic Vulnerability’ Exclusion criteria: − History of DSM‐IV‐TR affective or non‐affective psychotic episode; − Past exposure to antipsychotic medication; − Current DSM‐IV‐TR substance dependence; − Known severe/moderate intellectual disability (i.e., IQ < 50); and − Neurological disorder or any other medical condition presenting with psychiatric symptoms.
UHR measure	Comprehensive Assessment of At Risk Mental States (CAARMS)
UHR sample (number of participants and demographic details)	57 UHR‐P participants − 33 (57.9%) CHR‐P individuals met APS criteria and 23 (40.4%) met BLIPS criteria. − Schizotypal personality disorder was the most common diagnosis (*n* = 23; 40.4%), followed by brief psychotic disorder (*n* = 11; 19.3%) and borderline personality disorder (*n* = 10; 17.5%).
Substance use (specify types, measures etc.)	Sociodemographic/clinical schedule was completed at baseline which collected information on current substance misuse.
Outcome measures	As outcome assessment tool, the ‘Health of Nation Outcome Scale’ (HoNOS) was completed to assess levels of health and socio‐occupational functioning. The presence of suicidality (i.e., suicide/self‐harm thinking and behaviour) was defined by an at least HoNOS ‘Non‐accidental self‐injury’ item subscore of ≥ 1 (i.e., ‘…occasional thoughts about death or of self‐harm not leading to injury’).
Findings (results applicable to review question, number of participants included in analyses, analyses conducted)	While raw data were presented in tables, the study did not provide accompanying analysis or interpretive summary.
Quality appraisal (MMAT)	Screening questions (for all types) S1. Are there clear research questions? Rating: Yes Comment: The study's aims were clearly articulated, including unfavourable short‐term outcome indicators and associations with sociodemographic and clinical characteristics of the CHR‐P sample, which were relevant to the review objective. S2. Do the collected data allow to address the research questions? Comment: Yes Comment: Study included data on substance use and suicidality in the CHR‐P group. Quantitative descriptive 4.1. Is the sampling strategy relevant to address the research question? Rating: Yes Comment: The study employed non‐probability (convenience) sampling of CHR‐P adolescents and young adults enrolled in the first 5 years of activity of the ‘Parma At‐Risk Mental States’ (PARMS) program between January 2013 and December 2017. 4.2. Is the sample representative of the target population? Rating: Can't tell Comment: The study provided a clear description of the overall sample of 57 CHR‐P participants; however, details distinguishing substance users and non‐users (controls) were insufficiently described. 4.3. Are the measurements appropriate? Rating: No Comment: Substance use was assessed without the use of validated measurement tools. Additionally, no details provided on how substance use was assessed. 4.4. Is the risk of nonresponse bias low? Rating: No Comment: During the follow‐up, 13 (22.8%) CHR‐P participants dropped out. Kaplan–Meier analysis results confirmed a 1‐year cumulative incidence rate of 24.6%. In comparison with CHR‐P individuals who did not drop out, those with drop‐out condition showed shorter DUI at entry, lower baseline prevalence of previous specialist contact and antipsychotic prescription, lower acceptance of all PARMS psychosocial treatment, and higher baseline prevalence of BLIPS and emergency room/general hospital as source of referral. In Cox regression analysis, no statistically significant predictive factor was found. 4.5. Is the statistical analysis appropriate to answer the research question? Rating: Can't tell Comment: The study's research questions only partially aligned with the focus of this review, and statistical analyses were not reported for substance types.
Additional notes	Nil

### Study 8: Pfluger et al. (2025)

**Table:**

Source (author, year, country bibliographic reference)	Pfluger, J., Green, J. B., Qi, W., Goods, C., Rodriguez, J., West, M. L., Keshavan, M., & Friedman‐Yakoobian, M. (2025). Prevalence and Clinical Correlates of Suicidal Ideation and Attempts in Individuals at Clinical High Risk for Psychosis. *Early intervention in psychiatry*, *19*(1), e13633. https://doi.org/10.1111/eip.13633 Country: United States
Study design	Cross‐sectional
Sample source (study name, year)	CHR‐ P clinic at the Center for Early Detection, Assessment, and Response to Risk (CEDAR) between January 2017 and April 2025
Inclusion/exclusion criteria	Inclusion criteria: − Age 12–30 years; − Spoke English − Met broadly defined CHR‐ P criteria as assessed by the Structured Interview for Psychosis Risk Syndromes Exclusion criteria: − Intellectual disability, and prior history of experiencing fully psychotic symptoms as defined by the Presence of Psychotic Symptoms (POPS) criteria outlined in the Structured Interview for Psychosis Risk Syndromes.
UHR measure	Structured Interview for Psychosis‐ Risk Syndromes (SIPS)/Scale of Psychosis Risk Syndromes (SOPS)
UHR sample (number of participants and demographic details)	135 UHR‐P participants − A total of 194 clients completed an assessment between January 2017 and April 2022. Sixteen clients were removed from the analysis due to meeting criteria for a current or past full psychosis, 36 were removed for having no attenuated psychosis symptoms, and 7 were removed due to missing data, leaving a total of 135 clients. − Mean age of 17.7 (SD = 3.8), with a total of 53 (39.3%) cis‐ female, 57 (42.2%) cis‐ male clients, 5 (3.7%) transgender female clients, 3 (2.2%) transgender male clients, 12 (8.9%) other identity or non‐ binary clients, and 5 (3.7%) clients did not report gender identity. − Nine (6.7%) participants identified as Asian, 14 (10.4%) Black or African American, 13 (9.6%) Latinx, 71 (52.6%) White, 15 (11.1%) Bi‐ racial/Multiracial, 5 (3.7%) other, and 8 (5.9%) participants did not report racial identity. − The recoded gender identity variable included 53 (39.3%) cis‐ gender female, 57 (42.2%) cis‐ gender male, and 20 (14.8%) gender‐ expansive − 56 (41.5%) non‐ white and 71 (52.6%) white participants. − 88 (65.2%) of clients reported lifetime SI and 30 (22.2%) reported a lifetime SI
Substance use (specify types, measures etc.)	Alcohol Use Scale (AUS)/Drug Use Scale (DUS)
Outcome measures	Clinician recorded responses of SI ‘Have you had any thoughts of killing yourself?’ and lifetime responses of SA ‘Have you tried to end your life?’ The Screening for Risk of Harm (SRH) is a 16‐ item clinician administered interview of risk of harm to self and others developed for internal use at CEDAR.
Findings (results applicable to review question, number of participants included in analyses, analyses conducted)	While raw data were presented in tables, the study did not provide accompanying analysis or interpretive summary.
Quality appraisal (MMAT)	Screening questions (for all types) S1. Are there clear research questions? Rating: Yes Comment: The study's aims were articulated, including clinical correlates between SI and SA in a help‐ seeking clinical CHR‐ P sample, which were relevant to the review objective. S2. Do the collected data allow to address the research questions? Comment: Yes Comment: Study included data on substance use and suicidality in the CHR‐P group. Quantitative descriptive 4.1. Is the sampling strategy relevant to address the research question? Rating: Yes Comment: The study employed non‐probability (convenience) sampling of clients who completed baseline evaluations at CEDAR, which is a CHR‐ P clinic. 4.2. Is the sample representative of the target population? Rating: Can't tell Comment: The study provided a clear description of the overall sample of 135 CHR‐P participants; however, details distinguishing substance users and non‐users (controls) were insufficiently described. 4.3. Are the measurements appropriate? Rating: Can't tell Comment: Although the study reported using validated tools, that is, Alcohol Use Scale (AUS)/Drug Use Scale (DUS), only results from the AUS was explicitly presented. The study also reported on substance use disorders and a general ‘substance use scale’, but it is unclear whether these outcomes were derived from the DUS or combination of AUS and DUS. The lack of clarity raises concerns about how the validated tools were applied and how the results were reported, making it difficult to determine the appropriateness of the measurements. 4.4. Is the risk of nonresponse bias low? Rating: Yes Comment: Data was obtained from 135 clients and only 7 were removed due to missing data. 4.5. Is the statistical analysis appropriate to answer the research question? Rating: Can't tell Comment: The study's research questions only partially aligned with the focus of this review, and statistical analyses were not reported for substance types.
Additional notes	Nil

### Study 9: Preti et al. (2009)

**Table:**

Source (author, year, country bibliographic reference)	Preti, A., Meneghelli, A., Pisano, A., Cocchi, A., & Programma 2000 Team (2009). Risk of suicide and suicidal ideation in psychosis: results from an Italian multi‐modal pilot program on early intervention in psychosis. *Schizophrenia research*, *113*(2–3), 145–150. https://doi.org/10.1016/j.schres.2009.06.007 Country: Italy
Study design	Cross‐sectional
Sample source (study name, year)	Programma 2000
Inclusion/exclusion criteria	Inclusion criteria: − Patients aged 17–30; − First contact with any public mental health service from the catchment area for a first episode of psychosis (with DUP ≤ 24 months), or being referred to Programma 2000 with a suspected psychosis. − A score ≥ 12 on the ERIraos‐CL and evidence of positive symptoms on the BPRS– even if in an attenuated or subthreshold form– associated to signs of social withdrawal on the GAF. Exclusion criteria: − Past or present diagnosis of psychosis in the spectrum of schizophrenia or affective disorders
UHR measure	A score ≥ 12 on the ERIraos‐CL and evidence of positive symptoms on the BPRS
UHR sample (number of participants and demographic details)	81 in HRP group − Male/female ratio = 2.3 − Age at assessment did not differ by sex (males: mean = 22.0; SD = 3.7; females: mea*n* = 23.0; SD = 3.5)
Substance use	Not stated
Outcome measures	Reported by the patient at the assessment and/or by a family member consulted as a key informant (no discrepancy), or recorded during treatment, within the first year of follow‐up.
Findings (results applicable to review question, number of participants included in analyses, analyses conducted)	An enhanced risk of attempted suicide before enrolment was observed among those who met the criteria for substance abuse (AUC = 0.779, 95% CI: 0.546–1.00)
Quality appraisal (MMAT)	Screening questions (for all types) S1. Are there clear research questions? Rating: Yes Comment: The study's aims were articulated, including association between sociodemographic and clinical profile at assessment and suicidality at assessment or at follow‐up, which were relevant to the review objective. S2. Do the collected data allow to address the research questions? Comment: Yes Comment: Study included data on substance use and suicidality in the high‐risk group (HRP). Quantitative descriptive 4.1. Is the sampling strategy relevant to address the research question? Rating: Yes Comment: The study employed non‐probability (convenience) sampling where data were collected during the routine assessment of patients attending the Programma 2000. 4.2. Is the sample representative of the target population? Rating: Can't tell Comment: The study provided a clear description of the overall sample of all 81 patients in HRP group; however, details distinguishing substance users and non‐users (controls) were insufficiently described. 4.3. Are the measurements appropriate? Rating: No Comment: Substance use was assessed without the use of validated measurement tools. Additionally, no details provided on how substance use and abuse were assessed. 4.4. Is the risk of nonresponse bias low? Rating: Yes Comment: Data was obtained from all 81 patients in HRP group. 4.5. Is the statistical analysis appropriate to answer the research question? Rating: Yes Comment: The study reported that an enhanced risk of attempted suicide before enrolment was observed among those who met the criteria for substance abuse (AUC = 0.779, 95% CI = 0.546–1.00).
Additional notes	Nil

### Study 10: Taylor et al. (2015)

**Table:**

Source (author, year, country bibliographic reference)	Togay, B., Noyan, H., Tasdelen, R., & Ucok, A. (2015). Clinical variables associated with suicide attempts in schizophrenia before and after the first episode. *Psychiatry research*, *229*(1–2), 252–256. https://doi.org/10.1016/j.psychres.2015.07.025 Country: Turkey
Study design	Longitudinal
Sample source (study name, year)	First‐episode Schizophrenia Follow‐up Project at the Department of Psychiatry, Istanbul Faculty of Medicine
Inclusion/exclusion criteria	Inclusion criteria: − Patients aged 14–45; − Being in acute phase of their first psychotic episode, and being between age of 15 and 45. Exclusion criteria: − Past diagnosis of nonaffective possible psychosis; − Previous antipsychotic treatment more than 15 days
UHR measure	No UHR measure; Patients diagnosed as schizophrenia by means of the Structured Clinical Interview for DSM‐IV (SCID) then were re‐evaluated at a consensus meeting incorporating clinical and SCID data. Note by author: the period before first episode psychosis considered as prodromal phase
UHR sample (number of participants and demographic details)	172 patients
Substance use	Not stated how alcohol use was assessed
Outcome measures	Attempted suicide before admission, gathered from the patients themselves and their families.
Findings (results applicable to review question, number of participants included in analyses, analyses conducted)	We found that 29 of 172 patients (16.5%) attempted suicide before admission to our clinic. When we analysed suicide attempt before admission as a dependent variable in logistic regression analyses and alcohol/substance use appeared as an independent variables that significantly contributed to suicide attempts prior to admission.
Quality appraisal (MMAT)	Screening questions (for all types) S1. Are there clear research questions? Rating: Yes Comment: The study's aims were articulated, including variables associated with suicide attempts in schizophrenia before first episode, which were relevant to the review objective. S2. Do the collected data allow to address the research questions? Comment: Yes Comment: Study included data on substance use and suicidality in patients with first‐episode schizophrenia. Quantitative descriptive 4.1. Is the sampling strategy relevant to address the research question? Rating: Yes Comment: The study employed non‐probability (convenience) sampling where data were collected from patients that were admitted to the Department of Psychiatry, Istanbul Faculty of Medicine who were diagnosed as having schizophrenia. 4.2. Is the sample representative of the target population? Rating: Can't tell Comment: The study provided a clear description of the overall sample of the 172 patients; however, details distinguishing substance users and non‐users (controls) were insufficiently described. 4.3. Are the measurements appropriate? Rating: No Comment: Alcohol use was assessed without the use of validated measurement tools and no details provided on how alcohol use was assessed. 4.4. Is the risk of nonresponse bias low? Rating: Yes Comment: Data on attempted suicide before admission was obtained from all 172 patients. 4.5. Is the statistical analysis appropriate to answer the research question? Rating: Yes Comment: Suicide attempt before admission was analysed as a dependent variable in logistic regression analyses with alcohol/substance use appearing as an independent variables that significantly contributed to suicide attempts prior to admission.
Additional notes	Nil

### Study 11: Trujillo et al. (2025)

**Table:**

Source (author, year, country bibliographic reference)	Trujillo, T., Mirzakhanian, H., Addington, J., Bearden, C. E., Cannon, T. D., Cornblatt, B. A., Keshavan, M., Mathalon, D. H., Perkins, D. O., Stone, W., Walker, E. F., Woods, S. W., & Cadenhead, K. S. (2025). High rates of suicidality and parasuicidal behaviour in individuals at clinical high‐risk for psychosis: Implications for suicide risk assessment and suicide prevention. *Schizophrenia research*, *281*, 1–9. Advance online publication. https://doi.org/10.1016/j.schres.2025.04.030 Country: United States
Study design	2‐year longitudinal study
Sample source (study name, year)	NAPLS; Participants in this study were part of NAPLS3, a 9‐site, 2‐year longitudinal study.
Inclusion/exclusion criteria	Inclusion criteria: − Aged 12–35
UHR measure	CHR participants met the Criteria of Psychosis‐Risk Syndromes (COPS) through the Structured Interview for Psychosis‐Risk Syndromes (SIPS)
UHR sample (number of participants and demographic details)	710 CHR − Between the ages of 12 and 35 years old at baseline assessment
Substance use (specify types, measures etc.)	Substance use was examined both via DSM diagnosis with the SCID‐V as well as via the Alcohol/Drug Use Scale (AUS/DUS) and the Cannabis Scale.
Outcome measures	Past SI, plans, self‐harm, and attempts were assessed via the clinician‐ administered Structured Assessment of Violence Risk in Youth (SAVRY) scale and the Calgary Depression Scale for Schizophrenia (CDSS) at the baseline time point. The items on these scales assessing suicidality are shown in Table 1.
Findings (results applicable to review question, number of participants included in analyses, analyses conducted)	Alcohol (*p*'s < 0.005), tobacco (*p*'s < 0.02), cannabis (*p*'s < 0.01), and cocaine (*p*'s < 0.02; NS) use were all significantly associated with history of suicidality and self‐harm. SCID‐V diagnosis of alcohol use disorder was significantly associated with history of SI on the CDSS (*p* < 0.02) and diagnosis of cannabis use disorder was significantly associated with history of self‐harm or parasuicidal behaviour on the SAVRY (*p* < 0.04). However, amount of cannabis used over the past 6 months at baseline was not significantly associated with history of SI or self‐harm. Analyses of use of other substances were limited by the small number of participants reporting use of these substances; therefore, this data was not reported.
Quality appraisal (MMAT)	Screening questions (for all types) S1. Are there clear research questions? Rating: Yes Comment: The study's aims were articulated, including risk and protective factors associated with suicidal ideation and parasuicidal behaviour among individuals at clinical high‐risk (CHR) for psychosis, which were relevant to the review objective. S2. Do the collected data allow to address the research questions? Comment: Yes Comment: Study included data on substance use and suicidality in CHR group. Quantitative descriptive 4.1. Is the sampling strategy relevant to address the research question? Rating: Yes Comment: The study employed non‐probability (convenience) sampling where data were collected from 710 CHR participants as part of the part of NAPLS3, a 9‐site, 2‐year longitudinal study. 4.2. Is the sample representative of the target population? Rating: Can't tell Comment: The study provided a description of the 697 and 684 CHR participants with SAVRY and CDSS data respectively; however, details distinguishing substance users and non‐users (controls) were insufficiently described. 4.3. Are the measurements appropriate? Rating: Yes Comment: Substance use was examined both via DSM diagnosis with the SCID‐V as well as via the Alcohol/Drug Use Scale (AUS/DUS) and the Cannabis Scale. 4.4. Is the risk of nonresponse bias low? Rating: Yes Comment: Of the 710 CHR participants, data was obtained for 684 (96.3%) for CDSS suicidal ideation and 697 (98.2%) for SAVRY self‐harm. 4.5. Is the statistical analysis appropriate to answer the research question? Rating: Yes Comment: The study used one‐way multivariate analyses of variance and chi‐square tests to determine the relationship of alcohol, tobacco, cannabis, and cocaine use and suicidality as measured by CDSS and SAVRY.
Additional notes	Nil

### Study 12 Zuschlag et al. (2018)

**Table:**

Source (author, year, country bibliographic reference)	Zuschlag, Z. D., Korte, J. E., & Hamner, M. (2018). Predictors of Lifetime Suicide Attempts in Individuals With Attenuated Psychosis Syndrome. *Journal of psychiatric practice*, *24*(3), 169–178. https://doi.org/10.1097/PRA.0000000000000303 Country: United States
Study design	Secondary analysis
Sample source (study name, year)	Secondary analysis of a pre‐existing database of individuals with APS who were treated across the Medical University of South Carolina's (MUSC) inpatient and outpatient settings for a 5‐year period between January 1, 2006 and December 31, 2010.
Inclusion/exclusion criteria	Not specified
UHR measure	Met diagnostic criteria for attenuated psychosis syndrome (APS) as defined in the fifth edition of the Diagnostic and Statistical Manual for Mental Disorders (DSM‐5)
UHR sample (number of participants and demographic details)	Individuals meeting diagnostic criteria for APS (*N* = 152) were included in the data analysis
Substance use (specify types, measures etc.)	Tobacco use as recorded in database
Outcome measures	Lifetime suicide attempt
Findings (results applicable to review question, number of participants included in analyses, analyses conducted)	Of these 152 individuals, 40 (26.3% of our study population) had at least 1 lifetime suicide attempt. In univariate analyses, 8 predictors of interest were found to be statistically significant predictors of lifetime suicide attempts: tobacco use (35.3% vs. 19.5%; *p* = 0.030); A multivariate logistic regression model was then fitted for each predictor of interest, controlling for race, sex, and age in each model. After controlling for these factors, we still observed a statistically significant increase in lifetime suicide attempts among individuals with APS who had tobacco use (*p* = 0.039, OR = 2.266, CI: 1.042–4.929)
Quality appraisal (MMAT)	Screening questions (for all types) S1. Are there clear research questions? Rating: Yes Comment: The study's aims were articulated, including predictive factors associated with suicidal attempts and completed suicide among individuals with prodromal psychosis, which were relevant to the review objective. S2. Do the collected data allow to address the research questions? Comment: Yes Comment: Study included data on substance use and suicidality in those identified as having attenuated psychosis syndrome (APS) Quantitative descriptive 4.1. Is the sampling strategy relevant to address the research question? Rating: Yes Comment: The study involved a secondary analysis of a pre‐existing database of individuals with APS. 4.2. Is the sample representative of the target population? Rating: Can't tell Comment: The study provided a description of the 152 individuals with APS; however, details distinguishing substance users and non‐users (controls) were insufficiently described. 4.3. Are the measurements appropriate? Rating: No Comment: Tobacco use was assessed without the use of validated measurement tools. 4.4. Is the risk of nonresponse bias low? Rating: Yes Comment: Secondary analysis of a pre‐existing database of individuals with APS. 4.5. Is the statistical analysis appropriate to answer the research question? Rating: Yes Comment: A multivariate logistic regression model was then fitted for each predictor of interest, controlling for race, sex and age in each model. After controlling for these factors, we still observed a statistically significant increase in lifetime suicide attempts among individuals with APS who had tobacco use (*p* = 0.039, OR = 2.266, CI, 1.042–4.929)
Additional notes	Nil
